# Genome-wide identification of microRNAs involved in the regulation of fruit ripening and climacteric stages in melon (*Cucumis melo*)

**DOI:** 10.1038/s41438-020-0331-3

**Published:** 2020-07-01

**Authors:** Selinge Bai, Yunyun Tian, Chao Tan, Shunbuer Bai, Jinfeng Hao, Agula Hasi

**Affiliations:** Key Laboratory of Herbage and Endemic Crop Biotechnology, Ministry of Education, School of Life Sciences, Inner Mongolia University, 010070 Hohhot, China

**Keywords:** Fruiting, Next-generation sequencing, Transgenic plants

## Abstract

Fruit ripening is influenced by multiple plant hormones and the regulation of genes. However, studies on posttranscriptional regulators (e.g., miRNAs) of fruit growth and ripening are limited. We used miRNA sequencing and degradome methods to identify miRNAs and their target genes in melon (*Cucumis melo* cv. Hetao melon). A total of 61 conserved miRNAs and 36 novel miRNAs were identified from fruit growth, ripening, climacteric, and postclimacteric developmental stage samples, of which 32 conserved miRNAs were differentially expressed between developmental stage samples. Sixty-two target genes of 43 conserved miRNAs and 1 novel miRNA were identified from degradome sequencing. To further investigate miRNA influencing fruit ripening, transgenic melon plants overexpressing pre-cme-miR393 (cme-miR393*-*OE) were generated and characterized. The results showed that fruit ripening was delayed in cme-miR393-OE transgenic lines compared to nontransgenic fruits. The target of cme-miR393 was also identified, and the expression of *CmAFB2* was repressed in transgenic plants. These results provide evidence that miRNA regulates melon fruit ripening and provide potential targets to improve the horticultural traits of melon fruit.

## Introduction

Fleshy fruits are an important source of nutrition for humans and have great economic value. Based on the respiration rate of fruit ripening, fleshy fruit can be classified into two classes: climacteric and non-climacteric fruit^1^. Climacteric fruit, such as tomato, apple, banana, mango and melon, show a dramatic change in respiration accompanied by autocatalytic ethylene synthesis at the onset of fruit ripening^2^. In contrast, non-climacteric fruits, for example, strawberry, grape, litchi, and pea, exhibit a constant respiration rate and produce little or no ethylene^3^. In contrast to non-climacteric fruits, climacteric fruits are usually harvested before fully mature, ripen after harvest with ethylene synthesis and accumulation, and soon perish after the respiratory climacteric stage, which strongly affects their shelf life.

miRNA is a small regulatory molecule that widely exists in animal and plant species. In plants, miRNAs are typically 20-22 nt long and transcribed by RNA polymerase II to form primary miRNAs (pri-miRNAs), which are precursors of miRNAs^4^. Many plant miRNAs are conserved across species and play important roles in many processes, including plant growth and development, biotic and abiotic stress tolerance, and signal transduction^5^. Fruit development and ripening also depend on the regulation of miRNAs. Several miRNAs may be involved in fruit morphological development. For example, over/down expression of miR164^6^, miR156/miR157^7,8^, miR396^9^, and miR160^10^ will lead to abnormal fruit, such as fused carpels, reduced/changed fruit size, and shape. Fruit coloring and softening are also regulated by miRNAs. In tomato, miR858 negatively regulates anthocyanin biosynthesis by downregulating *SlMYB7-like* and *SlMYB48-like* transcripts^11^. In pear, miR397 can reduce the lignin content and stone cell numbers by downregulating the key lignin biosynthesis enzyme laccase (LAC)^12^. With the help of next-generation sequencing, it is possible to identify conserved miRNAs in model/nonmodel plants and predict novel miRNAs. Many plant species have been studied using next-generation sequencing, such as tomato, apple, melon, and litchi.

For a long time, tomato has been a model plant for studying fleshy fruit growth and ripening, especially for climacteric fruit. Tomato fruit belongs to berry fruit, in which the fruit of tomato is developed from the superior ovaries with one or more carpels. However, melon fruit is pepo fruit, whose fruits are derived from the inferior ovary with three carpels. This indicates that a different mechanism of fruit growth and ripening may involve^13^. Melon has a relatively small genome size and short life cycle, and these characteristics make melon an attractive model plant of the Cucurbitaceae family for studying fruit development and ripening^14^. However, studies on melon fruit ripening are limited. Zhang et al. identified the miRNAs involved in the regulation of fruit development in the nonrespiratory climacteric melon cultivar Hami melon^15^. Chayut et al*.* used bulk segregant transcriptome analysis to study the color variation of melon fleshy fruit^16^. To this end, in this study, we used Hetao melon as a model plant to study fruit growth and ripening.

Melon is also an important horticultural crop worldwide and has two respiratory climacteric and nonrespiratory climacteric types of fruit. In 2017, the entire world production of melon was more than 49 million tons, and China produced over one-third of the melon (FAO, http://www.fao.org/). Hetao melon has been cultivated in the Hetao area in the western part of the Inner Mongolia Autonomous Region for over 70 years and is still one of the main varieties. The melon is suitable for studying fruit growth and climacterics. The fruits of Hetao melon are small early maturing varieties (approximately 40 DAP). The fruit turns yellow, and the abscission zone (AZ) begins to form on the stalk of fruit when the fruit ripens. It has a typical climacteric phenotype with strong fragrance during the climacteric stage. After the climacteric stage, the fruit quickly softens and rots^17^. Studying the mechanism of melon growth, ripening and climacteric growth will provide a scientific basis for further improvement of the fruit.

In this study, we used samples of melon fruit of four typical developmental stages (growth stage (G), ripening stage (R), climacteric stage (C) and postclimacteric stage (P)) to identify the conserved and nonconserved miRNAs and novel miRNAs that might regulate melon fruit growth, maturity and climacteric development. Degradome sequencing was used to determine the target of the miRNAs and identify the miRNA-mRNA target pairs. In addition, overexpressing pre-cme-miR393 transgenic plants showed a delayed ripening time. This study will deepen our understanding of melon fruit development and the respiratory climacteric system.

## Results

### miRNA sequencing of melon fruit

**Fig. 1:**
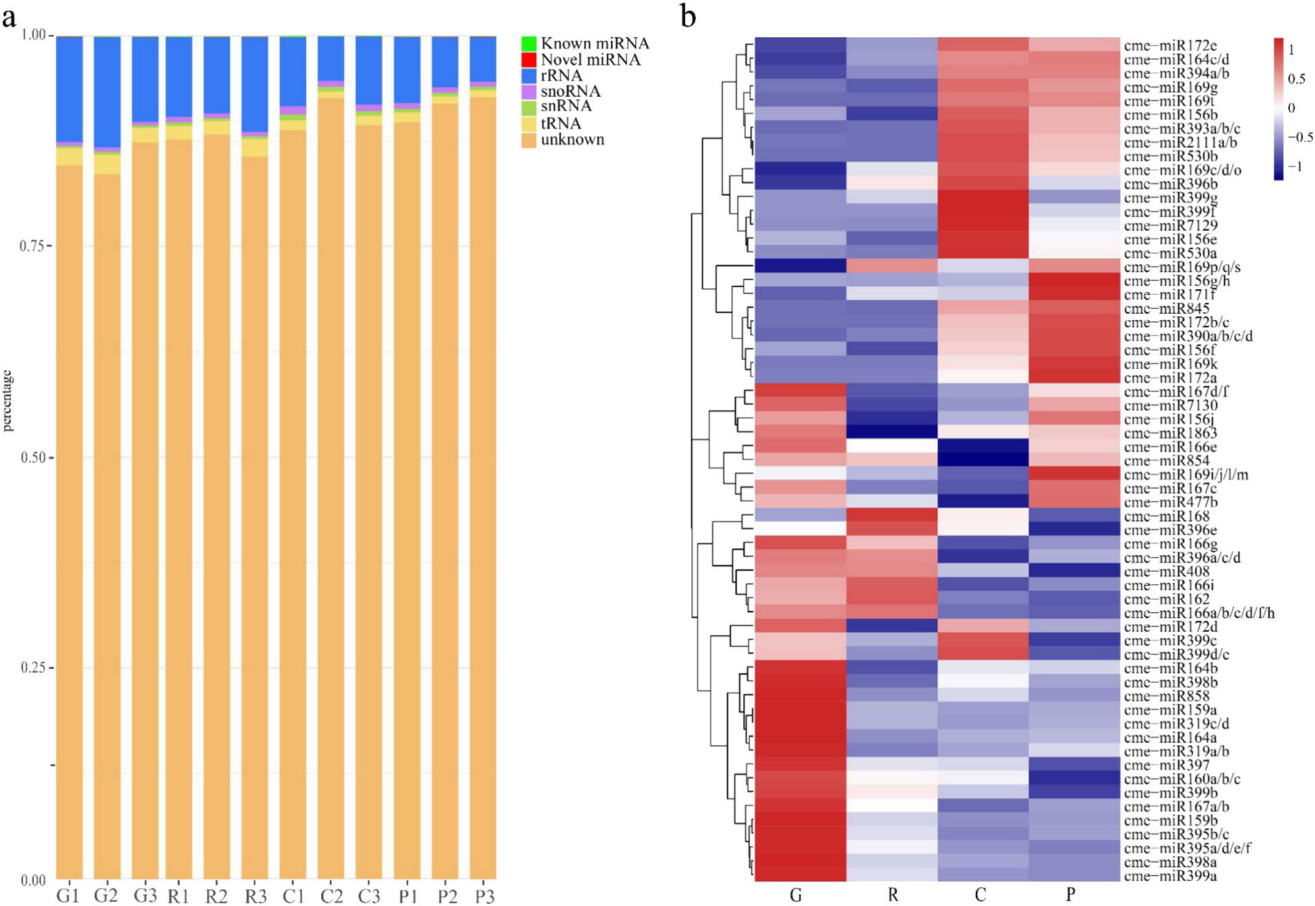
Annotation of small RNA unique reads and Heat map of conserved miRNA expression profile. **a** Annotation of Small RNA unique reads of the twelve libraries. **b** Expression profile and clustering of conserved miRNAs. Alphabet behind miRNA indicates the homolog of miRNA that has the same mature miRNA sequence. G growth stage, R ripening stage, C climacteric stage, P postclimacteric stage

miRNA sequencing was used to identify miRNAs involved in the processes of the melon fruit G stage to the P stage samples. On average, approximately 13.1 million raw reads were obtained from the 12 libraries of the four developmental stages of melon fruit. An average of approximately 10.2 million clean reads with lengths of 18–36 nt were generated (Supplementary Table 1); then, the repeat sequences of the clean reads were removed to obtain the unique reads (Supplementary Fig. 1). The unique reads were blasted to the Rfam 11 database to filter out noncoding RNAs (ncRNAs), rRNAs, tRNAs and snoRNAs. The remaining unique reads were then blasted against the miRbase 21 database to identify the conserved miRNAs. Most of the reads were not matched to any kind of known sRNA; the other large categories were rRNAs, tRNAs and snoRNAs, and miRNAs were the smallest category with low expression compared to other categories of sRNA (Fig. 1a). In this study, 61 miRNAs belonging to 29 miRNA families were found in the 12 libraries of melon fruit, and most of the miRNA families had at least one family member expressed in melon fruit (Supplementary Table 2).

### The expression characteristics of miRNAs in melon fruit

In general, the expression of miRNAs in melon fruit was not abundant. Twenty-nine miRNA expression levels were lower than 10 CPM, 16 miRNA expression levels were >10 CPM and <100 CPM, and 16 miRNA expression levels were higher than 100 CPM. Cme-miR159a was the most abundant miRNA, and the average expression was higher than 2000 CPM in four stage samples. Cme-miR159a was especially highly expressed in G stage samples and gradually downregulated (Supplementary Table 2). However, the average expression of cme-miR159b was much lower than that of cme-miR159a. The average expression of miRNAs can be generally divided into three groups: the first group is the miRNAs relatively highly expressed in at least one of the G and R stage samples, such as cme-miR159 and cme-miR162; the second group is miRNAs relatively highly expressed either in C or P stage samples, for example, cme-miR164d and cme-miR530b; the miRNAs relatively highly expressed in G and P stage samples and weakly expressed in the R and C stage samples belong to the last group (Fig. 1b). Differential expression analysis indicated that 14 miRNAs (7 upregulated and 7 downregulated) were differentially expressed in the fruit growth stage compared to the ripening stage (G vs. R) samples, 18 miRNAs (11 upregulated and 7 downregulated) were differentially expressed in the ripening stage compared to the climacteric stage (R vs. C) samples, and 4 miRNAs (1 upregulated and 3 downregulated) were differentially expressed in the climacteric stage compared to the postclimacteric stage (C vs. P) samples (Supplementary Fig. 2).

**Fig. 2:**
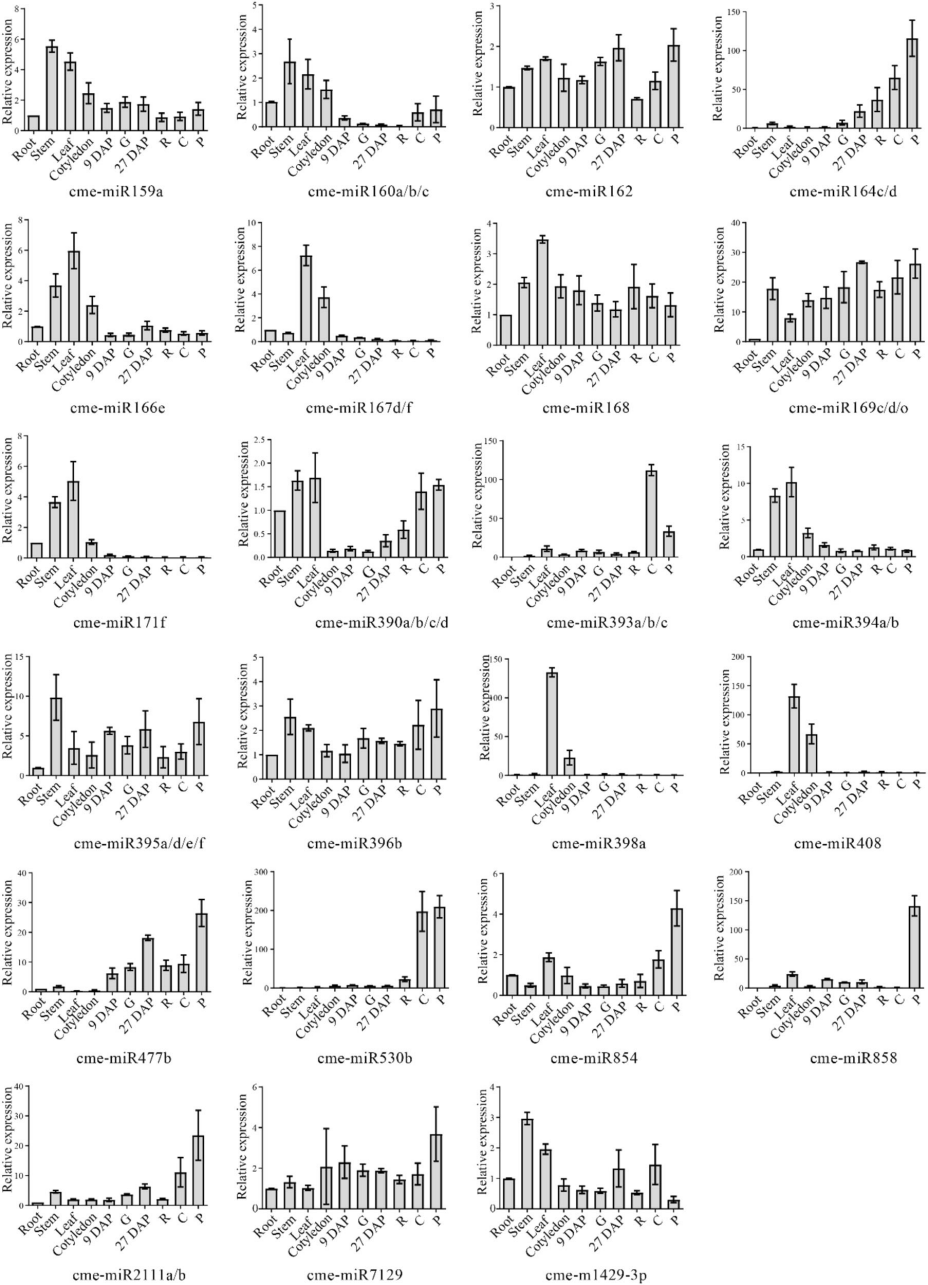
qRT-PCR validation of conserved miRNA and novel miRNA in different melon tissues and fruit developmental stages. DAP day after pollination, 9 DAP mesocarp of 9 DAP fruit, 27 DAP mesocarp of 27 DAP fruit, G growth stage, R ripening stage, C climacteric stage, P postlimacteric stage. Bars indicate miRNA abundance from the qRT-PCR results. Experiments were performed in three biological replicates. Error bars represent the mean ± SD of three biological replicates

To explore the expression of miRNAs in different melon tissues and validate the miRNA sequencing results, qRT-PCR was employed. Four melon tissue (root, leaf, stem, cotyledon) samples and fruit samples from 9 DAP, 27 DAP and the four sequencing fruit stage samples were used. In total, 23 miRNAs (22 conserved miRNAs and one novel miRNA) from different melon miRNA families were analyzed (Fig. 2), and the chosen miRNAs were highly expressed among the families. Most of the expression of miRNAs has a similar trend to the miRNA sequencing results. Six miRNAs (cme-miR164c/d, cme-miR393a/b/c, cme-miR477b, cme-miR530b, cme-miR858, cme-miR2111a/b) were specifically highly expressed in the fruit mesocarp.

### Novel miRNAs in melon fruit

Due to the low accuracy and high false positive rate of novel miRNA prediction, we predicted the novel miRNAs generally following the new criteria of miRNA annotation. We also used our pre-experiment miRNA sequencing data (growing in different years) together to predict novel miRNAs. Finally, 36 sequences were identified, the length of mature novel miRNAs ranged from 20 nt to 24 nt, the average MFEI of the pre-miRNAs was 1.16, and genome mapping of their premature miRNA sequences revealed that their average length was 84 nt (Table 1). RNA folding analysis using premature sequences of the novel miRNA showed that they have a single miRNA:miRNA* duplex and no secondary stem or large loops interrupting the duplex. The novel miRNA was named by the novel miRNA prediction software according to the predicted order of the novel miRNA candidates.

**Table 1:** Table of novel miRNAs

ID	Mature-seq	Len	MFE	MFEI
cme-m0240-3p	TATCAAAGTTACGCGATTGTTT	22	27.3	1.24
cme-m0426-3p	TAACGATCGTTTAACATAACTAAA	24	30.2	1.59
cme-m0519-3p	ATCTCACGGCGTCGGGAAAGCCT	23	33.3	0.85
cme-m0669-5p	AGACTGTGTTAGACTACTATGTGT	24	24.6	0.88
cme-m0761-5p	TAGCCAAATCTAAACCATTGTTTT	24	29.6	1.18
cme-m1117-5p	ATTTGGATCGACTTTCTAAGTGTT	24	27.8	0.93
cme-m1125-5p	TCTTTTGATTGTAGGACAATTTTT	24	20.1	0.91
cme-m1142-5p	AAGGATAAAAGGAAAAAAAGAGAA	24	19.7	1.16
cme-m1429-3p	TGCCAAAAGAGACTTGCCCTG	21	42.3	1.18
cme-m1690-3p	TTAAAATCGACATTAAACATCCGC	24	40.9	1.41
cme-m1707-3p	AACGATCGTGTAGATGATGATACA	24	31.1	0.89
cme-m1720-3p	TAGAAGAATATCATTGATAGTAGC	24	42.7	1.53
cme-m1907-3p	CTAAAAATTCTTGACGTTTT	20	28.5	1.19
cme-m2133-3p	AAACGATCGTTTAGTTATAA	20	20.7	1.15
cme-m2160-3p	AACGATCGTTTAGTGAATAGAAGT	24	20.6	0.98
cme-m2193-5p	GTGTTCTTGAAGTTGGAGTCTTTG	24	35.1	0.88
cme-m2518-5p	CGGTTTTAAACTGTTAAGAAT	21	27.2	0.91
cme-m3092-5p	AACGATCGTTTAGTGAATGAAATG	24	25.4	1.02
cme-m3595-5p	ATGATTGTTTAGATTTGGCTACA	23	22.6	0.98
cme-m3618-3p	TTGTGGACCTAGTTGACGAGTGC	23	41	1.14
cme-m3807-3p	CACGTGCTCCCCTTCTCCAAC	21	38.7	0.90
cme-m3823-3p	CAGTTTTAAAACGTCAAGAATTT	23	34.3	1.27
cme-m3880-3p	AACAATCGTGTATCAATATTTAAA	24	19.9	1.05
cme-m3941-3p	TAAACGATCGCTTAAACATATA	22	24.4	1.22
cme-m4047-5p	CTATCCAAATCTAAATGATATTT	23	18.2	0.87
cme-m4401-5p	AAAGGAAAAAGAGGGAAAATGAAA	24	28.1	1.12
cme-m4707-5p	GAGACATAAACATGGATTAACACT	24	40.2	1.75
cme-m4758-3p	AAGGATAATTTTCATAAATGTAAC	24	29.4	1.84
cme-m4873-5p	TATGTACGACGTCGGGAGAAGTT	23	30.9	0.86
cme-m4933-5p	AAGTGGATCTTTAATGTCGGTTTT	24	29.8	1.24
cme-m5360-3p	AAAACCGACATTAAACATCTTGCT	24	63.8	1.93
cme-m5388-5p	TTTGTTACATGGTCTATTAGTGAT	24	25.7	1.12
cme-m5563-5p	CGGTTGAAAACTGACATTAAAGGA	24	33.7	1.16
cme-m5576-5p	AATATGGAAAGGAAAAGTGAGACT	24	18.1	0.86
cme-m5814-5p	CTTTGAAAGATGCTAGTGAATACC	24	32.1	1.23
cme-m5892-5p	AGTCTTTAATGTCGGTTTTAAACC	24	47.4	1.58

**Fig. 3:**
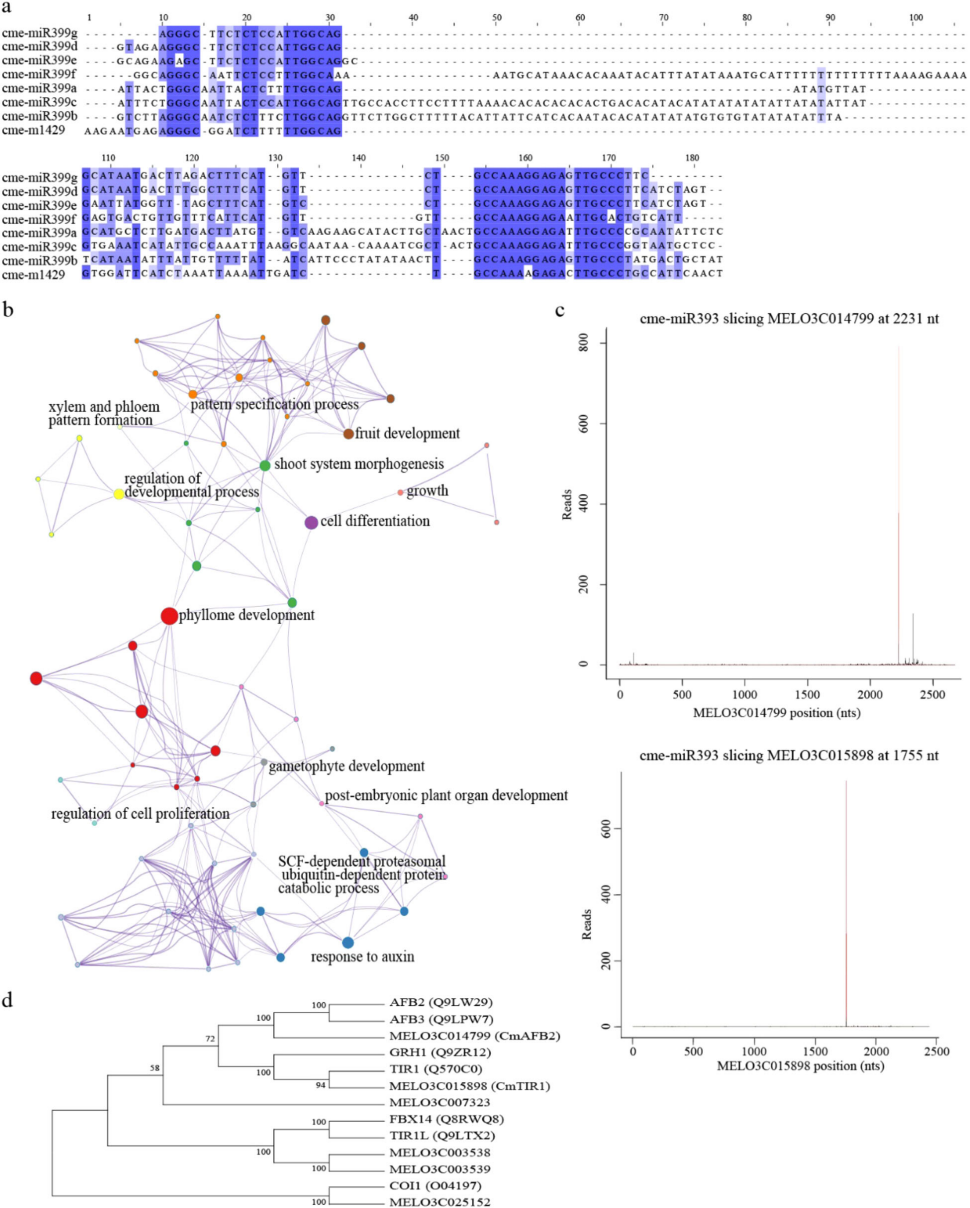
Novel miRNA sequence alignment, target gene GO enrichment, and target gene analysis of miR393. **a** Sequence alignment of cme-m1429 and cme-miR399 family members. Conserved sequences are shown in blue. **b** GO enrichment of target genes of conserved miRNA. The enriched GO terms of the same color indicate the same cluster, and the size of the node represents the number of input genes falling into that term. **c** T-plot of target genes of cme-miR393. The red line indicates the cleavage site of the target gene. **d** Phylogenetic analysis of the TIR1/AFB family genes of melon and *Arabidopsis*

The mature novel miRNA sequences were then blasted to miRBase data for a homologous miRNA search. Cme-m1429-3p was found to be similar to the cme-miR399 family. Multiple sequence alignment of the cme-miR399 family and cme-m1429-3p pre-miRNA sequence (cme-m1429) showed that both cme-m1429-3p and cme-m1429-5p sequences were conserved to the miR399 family (Fig. 3a).

### Target analysis of miRNA in melon fruit

In plants, miRNAs usually function as leading RNAs and promote the cleavage/inhibition of target mRNAs. Therefore, miRNA target recognition is helpful for understanding miRNA function. The prediction of miRNA targets is usually based on base pairing with complementary sequences between the query miRNA and target genes. Using psRobot with a score <= 2.5, 224 target genes were predicted for 67 miRNAs, including 59 conserved miRNAs and 8 novel miRNAs. To obtain more reliable miRNA targets, degradome sequencing was performed to detect cleavage mRNA in vivo by using PAREsnip^18^. Target genes of degradome sequencing can be classified into five categories (from 0 to 4) based on the hit of degradome fragments. The lower category indicates more hits of degradome fragments on the cleavage site. We chose category 2 as a threshold, which means the hit of degradome fragments on the cleavage site is above the average fragments. The results indicated an association between 48 miRNAs and their 60 target genes (Supplementary Table 3). Forty-seven target genes showed overlap between PAREsnip and psRobot. In addition, some single miRNAs target several genes, such as cme-miR156 and cme-miR164; in contrast, one gene could also be regulated by two or more miRNAs, for example, MELO3C019923, which is targeted by cme-miR159 and cme-miR319. Among the 60 target genes, 35 genes were transcription factors belonging to 11 gene families, including *AP2*, *ARF*, *GRF*, *TCP*, and *NAC*. Gene ontology (GO) analysis revealed that these target genes were enriched in biological processes such as fruit development (GO:0010154), response to auxin (GO:0009733), and cell differentiation (GO:0030154). (Fig. 3b). Target genes of differentially expressed miRNAs were used for GO analysis. There were 5 GO terms enriched in G vs. R stage samples and 7 GO terms enriched in C vs. P stage samples. The GO terms response to auxin (GO:0009733) and seed development (GO:0048316) were significantly enriched in the C vs. P stage samples.

Hormones play a key role in fruit ripening, such as ethylene and auxin. However, our results show that target genes of miRNAs were enriched in auxin signaling, involving 16 target genes and 8 miRNAs. Among the 8 miRNAs, three miRNAs (cme-miR164, cme-miR168, cme-miR394) were upregulated in G vs. R stage samples. Two NAC domain protein family genes (*CmNAC21* and *CmNAC92*) were the target genes of cme-miR164. *CmNAC21* and *CmNAC92* were homologs of *AtNAC1* and *AtNAC6*, respectively. Correlation analysis using our transcriptome data (data not published) and the miRNA expression data shows that the expression of *CmNAC21* and *CmNAC92* was significantly negatively correlated with the expression of cme-miR164. However, argonaute 1 (AGO1) and F-box family protein 6 (FBX6), which were the target genes of cme-miR168 and cme-miR394, respectively, did not exhibit a negative correlation. Another three miRNAs (cme-miR166, cme-miR167 and cme-miR393) were differentially expressed in R vs. C stage samples. Cme-miR393 was upregulated in R vs. C stage samples and targeted *CmTIR1* and *CmAFB2* genes, and their expression exhibited significant negative correlations. Cme-miR166a/g/i and cme-miR167a/b were downregulated in R vs. C stage samples and did not show significant negative correlations to their target genes *HB-8* and *ARF8*. Cme-miR160a/b/c targeted three ARF family genes (*CmARF16*, *CmARF17*, and *CmARF18*), but the expression of cme-miR160a/b/c in all melon fruit was low.

### cme-miR393 characteristics and its target genes

In melon, three miR393 precursors with typical stem-loop structures have been characterized, and their mature miRNA sequences are identical, named cme-miR393a, cme-miR393b and cme-miR393c, which are located on chromosomes 10, 3, and 6, respectively. Analysis of degradome data identified a significant cleavage site at 1755 bp and 2231 bp of *CmTIR1* (MELO3C015898) and *CmAFB2* (MELO3C014799). The target genes *CmTIR1* and *CmAFB2* belonged to category 0, having only one peak, and the cme-miR393-mediated cleavage site matched the peak (Fig. 3c). However, except *CmTIR1* and *CmAFB2*, no more genes were found with the cleavage site of cme-miR393 from the degradome data.

**Fig. 4:**
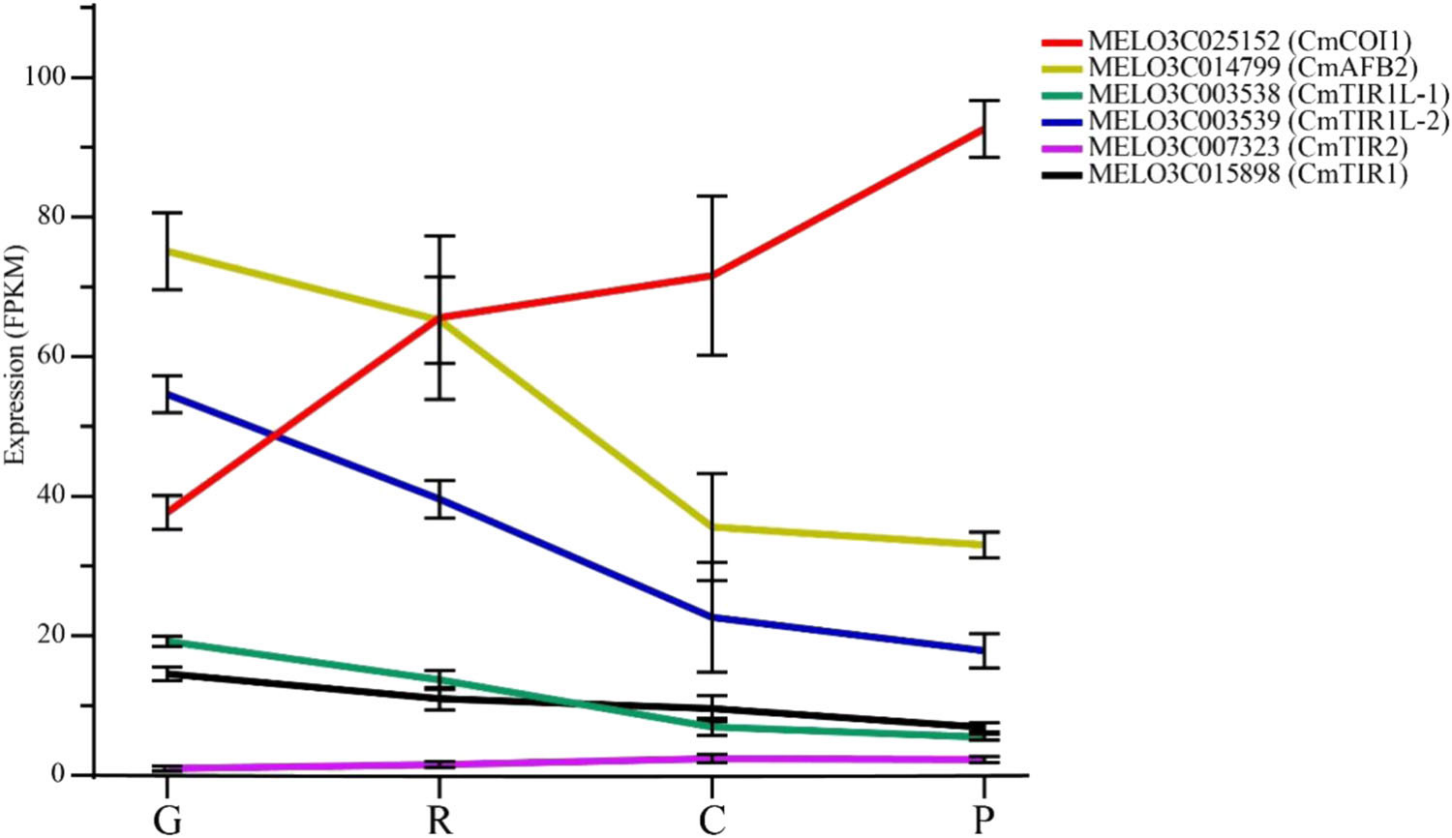
The expression of melon TIR1/AFB family genes. G growth stage, R ripening stage, C climacteric stage, P postclimacteric stage. Different genes are shown in different colors; error bars represent the mean ± SD of three biological replicates

In plants, the SCF^TIR1/AFB^ complex binds to auxin and promotes the ubiquitin-based degradation of AUX/IAA proteins, releasing ARF proteins to initiate downstream gene transcription^19^. To further understand the relationship of cme-miR393 and target genes, genome-wide identification of the TIR1/AFB family was performed using the PFAM (PF18791) database (Supplementary file 1). Six genes of the TIR1/AFB family were identified, all of which (MELO3C025152, MELO3C014799, MELO3C003538, MELO3C003539, MELO3C007323, and MELO3C015898) harbored a transport inhibitor response 1 protein domain. Phylogenetic analysis using melon and *Arabidopsis* TIR1/AFB family genes indicated that melon TIR1/AFB family genes are orthologous to *Arabidopsis* TIR1/AFB family genes (Fig. 3d)^20^. The expression profile of the TIR1/AFB family genes of transcriptome data showed that MELO3C007323 was expressed at low levels in the four stage samples, and the expression of MELO3C014799, MELO3C003538, MELO3C003539, and MELO3C015898 showed a similar expression pattern that was downregulated from G to P stage samples. However, MELO3C025152 exhibited an opposite expression pattern to the other four genes (Fig. 4).

### Overexpression of cme-miR393 in melon retarded fruit ripening

**Fig. 5:**
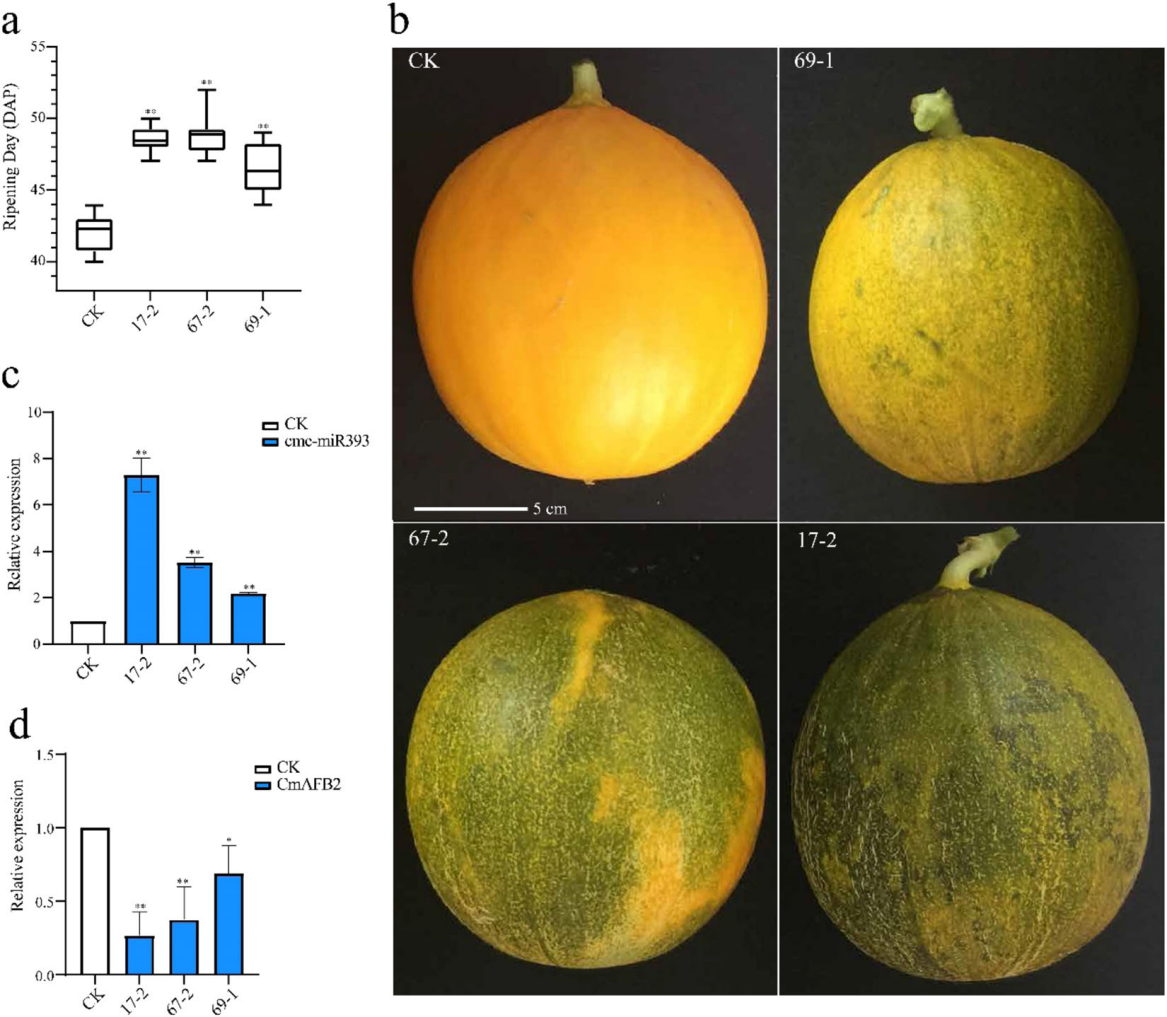
Phenotype and expression analysis of cme-miR393 overexpression transgenic lines. **a** Comparison of fruit ripening days of transgenic lines and nontransgenic melon. Error bars represent ± SD of the 3 transgenic lines with 10 replicates each. **b** Fruits of nontransgenic and transgenic lines. Fruits harvested when nontransgenic fruits ripened (43 DAP). Scale bar = 5 cm. **c** Relative expression of cme-miR393 in mesocarps at the ripening stage of fruit. **d** Relative expression of CmAFB2 in mesocarps at the ripening stage of fruit. Data represent the mean ± SD of three biological replicates. CK: control group of nontransgenic melon fruits; 17-2, 67-3, 69-1: fruits of cme-miR39 3 overexpression transgenic lines (Student′s *t-*test; *p < 0.05; **p < 0.01)

To investigate whether cme-miR393 affects melon fruit ripening, we generated transgenic melon plants overexpressing cme-miR393. Since the mature forms of cme-miR393 are identical, we chose pre-cme-miR393a for overexpression under the cauliflower mosaic virus (CaMV) 35S promoter. The pollen-tube pathway method was used to transform pre-cme-miR393a into melon. Transgenic melons were grown in a greenhouse, and fruit phenotypes were observed in the third generation of transgenic plants in comparison with the control (nontransgenic) plants. Analysis of the ripening time of control plants shows that the average fruit ripening day was 42.3 days after pollination (DAP). However, the transgenic plants exhibited retarded fruit ripening time, and the average fruit ripening days in the three transgenic plant lines 67-2, 69-1, and 17-2 were 48.5, 48.9, and 46.4 DAP, respectively. Simultaneously, fruits of control and transgenic plant lines showed obvious phenotypic differences. At approximately 43 DAP, the majority of the control fruits turned full yellow, and AZ was formed on the stalk of the fruits. The transgenic fruits entered the breaking color stage, and the ripening day was significantly different from the control fruits (Fig. 5a, b). qRT-PCR assays were performed to analyze the expression of cme-miR393 and target genes. A significant upregulation of cme-miR393 was found in the transgenic plant lines (17-2, 67-2, and 69-1), and *CmAFB2* was downregulated (Fig. 5b, c). The same analysis could not be performed for *CmTIR1*, as there was no full-length gene sequence information available for which appropriate primers could be designed. Moreover, melon fruit weight, soluble solids content, and mesocarp firmness between the late-ripening transgenic fruits and the control fruits were studied. However, no significant differences were found between the control and transgenic fruit at the same developmental stages. This suggests that overexpression of cme-miR393 will not affect fruit weight, soluble solids content and mesocarp firmness in the same developmental stages.

## Discussion

### The sequencing data of miRNA

To investigate miRNAs involved in fruit growth, ripening and climacteric development, sRNA-seq was used. We constructed 18 sRNA libraries from four different fruit developmental stage samples. In 2015, six libraries of G (18 DAP) and R (36 DAP) stage samples were constructed and sequenced. The next year, 12 libraries from G to P stage samples were constructed and sequenced. Comparing the two years of sequencing results, there was no significant difference in the expression of miRNAs; however, the unannotated sequences displayed an obvious difference in the number of sequences. Zhang et al*.* used miRNA sequencing to study miRNA expression in Hami melon fruit^15^, but the expression of conserved miRNAs and differentially expressed miRNAs was different from our results. This situation may be due to first, the different varieties of melon we used; second, Hetao melon is a typical climacteric fruit, while the Hami melon studied by Zhang et al*.* is a non-climacteric fruit. The results suggest different regulation of miRNAs in different varieties of melon and differences between climacteric and non-climacteric fruit.

### Novel miRNA annotation

In recent years, high-throughput sequencing technology has been widely used to identify conserved and novel miRNAs involved in the regulation of plant development. Many conserved miRNAs were identified from different species. In this study, we annotated 36 sequences to novel miRNAs based on the new criteria for plant miRNA annotation. Although more detailed criteria for plant miRNA annotation have been provided, novel miRNA annotation is still challenging. According to our sequencing data, the conserved miRNAs showed a similar expression pattern between the pre-experiment and experimental data. However, the pre-experiment and experimental data exhibit a great difference in unannotated sequences. Lower than 5% of the unannotated sequences were found in both data sets, and approximately half of the sequences were only found in one of the libraries. In addition, most of the sequences (over 70%) were also expressed at low levels. The data indicate that most of the unannotated sequences may come from RNA segments, only a small portion of the sequences may belong to miRNA, and most of the unannotated sequences lack repeatability. Therefore, the repeatability of unannotated sequences in different sequencing libraries or even in a different batch of experiments may provide a more accurate annotation of novel miRNAs.

### miRNA in fruit growth, ripening, and climacteric

Flowering plant fruits are often defined as structures derived from a mature ovary containing seeds or as the dispersal unit. The gynoecium is in the center of the flower, which is derived from the fusion of carpels and plays a key role in fruit formation. The hypothesis that carpels evolutionarily originated from leaves for more than a decade^21^. A good amount of evidence from different plants supports this hypothesis, including miRNAs. In this study, four miRNAs (cme−miR159a/b, cme−miR319a/b/c/d, cme−miR398a, and cme−miR7130) and five miRNAs (cme−miR2111b, cme-miR393a/b/c, cme-miR396b, cme−miR530b, and cme−miR7129) were differentially and highly expressed in the G and C stages, respectively (Supplementary Fig. 2). These results indicate that these miRNAs may participate in the regulation of fruit growth in the G or C stages. Ripening inhibitor (*rin*) mutants are useful in understanding the fruit ripening process. In tomato, the expression of miR159 is regulated by ethylene and significantly decreased in *rin* mutant tomato fruit from the immature green (IM) stage to the yellow ripe (YR) stage^22^. A similar expression pattern of cme-miR159a/b was found in melon fruit, in which the expression of cme-miR159a/b decreased from the G (green color) stage to the C (yellow color) stage, illustrating a similar regulation pattern in melon fruit. miR319 shows tissue-specific expression and decreases *TCP* gene expression to regulate leaf elongation and cell expansion in *Arabidopsis*^23^ Studies in tomato showed that downregulation of miR396 resulted in an increase in the size and cell size of the sepal and ultimately resulted in the production of larger fruit^24^. In *Arabidopsis*, miR172c expression is activated by ARF8, and miR172c downregulates *AP2* expression, thereby promoting *Arabidopsis* fruit valve elongation^25^. According to our results, the expression of miR172 was limited in the four fruit stage samples, suggesting that miR172 may function in the early fruit developmental stage. Five miR156 members were detected, while only cme-miR156j was highly expressed. miR156 targets several SQUAMOSA PROMOTER BINDING PROTEIN-LIKE (SBP/SPL) family genes. Overexpression of miR156b in tomato not only resulted in more carpels but also the fusion of multiple carpels and the formation of differentiated tissues in ovules. miR399 may be involved in fruit quality. In maize, miR399 plays a crucial role in the integration of sucrose sensing and photoperiodic responses^26^. In strawberries, overexpression of miR399 influences fruit quality by improving the soluble sugar, soluble solid and vitamin C content^27^. On the other hand, the differences in miRNA family member expression also indicate a tight regulation of miRNA. The expression of cme-miR159a was much higher than that of cme-miR159b, and a similar situation was also found in the cme-miR164, cme-miR166 and cme-miR399 families. This situation also suggests that different cis-regulatory elements may exist on the promotor region of miRNAs. Fruit growth and ripening may be involved in a complex miRNA regulation network, and miRNA can be a potential target for improving fruit quality, but this needs to be further investigated.

To date, it remains unclear why some fruit will climacteric after harvest and the detailed mechanism of fruit climacteric. When fruit climacteric happens, the expression of ethylene is increased, a plausible hypothesis that ethylene and ACC promote and accelerate AZ formation and trigger a branch of ripening-related gene expression^28^. Hetao melon is a kind of climacteric melon. Colombié et al*.* used constraint-based modeling to study the climacteric in tomato fruits and found that starch and cell wall degradation fuels the respiration climacteric of tomato fruit, enabling rapid reprogramming of metabolism at ripening^29^. In this paper, we attempted to identify miRNAs involved in the regulation of climacteric processes. Eighteen known miRNAs were differentially expressed in the R to C stage, 14 in the G to R stage and 4 in the C to P stage. Ethylene has been considered a pivotal gas hormone in fruit ripening and promotion of climacteric. Although there were miRNAs upregulated in the C stage, such as cme-miR399g, we did not find any miRNAs that directly target genes that function in ethylene synthesis and signaling transduction. We postulated that miRNAs may regulate ethylene signaling through indirect or novel miRNAs and that auxin may have crosstalk with ethylene. Auxin inhibits ripening via ARFs and ERFs and has antagonistic effects with ethylene on fruit ripening^30^. miRNAs may be involved in this regulation process, for example, several miRNAs targeting different ARFs in melon, such as miR160, miR167, and miR390. Our results indicate that cme-miR167b was significantly downregulated in the C stage compared to the R stage, and cme-miR160c was also downregulated; however, the downregulation was not significant. Ming et al*.* studied the miRNAs that respond to ethylene treatment in banana and found that miR167 is downregulated after ethylene induction^31^.

### Auxin and miRNAs in fruit ripening

miRNAs recognized as genetic and epigenetic regulators have attracted increasing attention. Chen et al*.* summarized the miRNAs that regulate the horticultural traits of plants^32^. Plant hormones play important roles in plant fruit growth and development. Ethylene is instrumental in climacteric fruit ripening, and auxin also plays an unneglectable role in fruit ripening^33^. Auxin response genes were enriched in the GO analysis of the target genes of miRNAs. Cme-miR393 targeted *CmTIR1* and *CmAFB2*, cme-miR160 and cme-miR167 targeted several *ARF* genes. *CmTIR1* and *CmAFB2* are auxin receptor genes, and we identified the gene members of the TIR1/AFB family in the melon genome. The results indicate that melon has more than one *TIR1/AFB* gene. This situation is not uncommon in other species; for example, there were six members in rice, three members of plum, and only two members in cucumber^34–36^. Blast analysis suggested that *CmTIR1* and *CmAFB2* are orthologs of *AtTIR1* and *AtAFB2* and may have a parallel function in plant development.

In plants, TIR1 protein function has been widely studied, and these results suggest an important role of TIR1 in a wide range of developmental processes; however, the study of TIR1 in fruit ripening is still limited. In tomato, transgenic plants overexpressing *SlTIR1* displayed a severely dwarfed phenotype and altered leaf morphology, and tomato fruit exhibited early fruit development and formed parthenocarpy fruit^37^. Overexpression of *PsTIR1* in pea also leads to early fruit development^38^. The miR393-TIR1 homolog regulation module has been extensively studied, including salt and drought tolerance, nodulation, tillering, fruit set, etc. In rice, overexpression of miR393 leads to downregulation of *OsTIR1* and *OsAFB2*, resulting in more tillers, early flowering and less tolerance to salt and drought stress^39^. In addition, pleiotropic effects of plant development were induced by overexpression of a miR393-resistant form of *TIR1*, leading to inhibition of primary root growth, overproduction of lateral roots, altered leaf phenotype and delayed flowering^40^. In this study, we explored the effect of miR393 on fruit ripening. *CmTIR1* and *CmAFB2* were identified as the target genes of cme-miR393, and the expression of *CmAFB2* was dominant in the early melon fruit development stage. Meanwhile, the expression of cme-miR393 was low in the G and R stage samples and then increased in the C and P stage samples. Illustrating that there may be a miR393-AFB regulation module in the melon fruit ripening process. However, it has been reported that miR393 is highly expressed at anthesis and then gradually downregulated^41^. Transgenic plants of cme-miR393-OE displayed retarded ripening time, and the expression of *CmAFB2* was also downregulated. These results agree with previous findings that overexpression of *TIR1* leads to early fruit development. Analysis of transcriptome data revealed that five of the six *TIR1/AFB* family genes were expressed between 10 and 100 FPKM (Fig. 4). *CmTIR1* (MELO3C015898), *CmAFB2* (MELO3C014799) and two other genes (MELO3C003538 and MELO3C003539) exhibited an obvious downward trend from G to P stage samples. The four genes with similar expression trends may indicate a functional redundancy in melon fruit ripening. *CmAFB2* is probably the most important gene in terms of the expression of these four genes. However, the expression of MELO3C025152 rapidly increased in R stage samples compared to the expression of the other four genes. This result may indicate the regulation changes of melon fruit during ripening and suggest that MELO3C025152 may function predominantly in the latter stages. According to phylogenetic analysis, MELO3C025152 is homologous to *Arabidopsis COI1*. In *Arabidopsi*s, *COI1* is a core component of jasmonate signaling and affects growth, metabolite production and cell wall protein composition^42^. According to Fig. 4, the opposite expression of MELO3C025152 and *CmAFB2* may indicate the transformation of the auxin pathway to the jasmonate pathway during fruit ripening. These findings provide evidence that the miR393-AFB2 regulation module affects melon fruit ripening and may be a novel regulation mode of fruit ripening.

## Materials and methods

### Plant materials

Melon (*Cucumis melo* cv. Hetao melon) plants were grown in a greenhouse in Dengkou County (N40°19′46.07″, E107°0′11.46″), Inner Mongolia Autonomous Region, China, in 2016. The flowers were self-pollinated, the pollination time was recorded, and the date of pollination was recorded as 0 days after pollination (DAP). Only one fruit was kept for each melon plant. Then, fruits in the G stage (18 DAP) and R stage (36 DAP) were harvested, and 5 g of mesocarp was frozen immediately in liquid nitrogen and kept in a refrigerator at −80 °C for later analysis. For each sample, 3 replicates were obtained from different plants.

To obtain climacteric melon fruit, we chose melon fruits whose color had just turned to full yellow (approximately 40 DAP). Then, the fruits were harvested, the respiration rate of the fruit was immediately measured by an infrared CO_2_ analyzer (Top Instrument Ltd., Zhejiang, China), and the respiration rate was measured every 2 h until the concentration of CO_2_ quickly increased^43^. Then, the mesocarp of the melon fruit was frozen in liquid nitrogen immediately. To obtain postclimacteric fruit, when the concentration of CO_2_ peaked, the respiration rate of the fruit was measured until approximately 48 h, and the concentration of CO_2_ returned to the starting level; therefore, postclimacteric fruit samples (approximately 42 DAP) were obtained. Fruit firmness was tested using a fruit penetrometer (EFFEGIDI, FT 011, Italy). Four symmetry points were measured at the vertical section of the mesocarp. Soluble solids content was tested following the instructions (Pocket refractometer: ATAGO, Cat. No. 3830, Japan).

For cultivation of melon seeds, plump seeds were selected, soaked in 75% alcohol to sterilize for 2 min, and then disinfected in 0.1% HgCl_2_ for 8 min. Seeds were cultured in 1/2 MS medium in the artificial climate incubator: 16 h light/8 h dark culture, 24–26 °C, 60% relative humidity. After 5 leaves were grown, 1 g of the roots, stems, leaves and cotyledon tissues were separated, immediately frozen in liquid nitrogen and preserved in the refrigerator at −80 °C, and 3 replicates were obtained from different plants.

In 2015, we also grown melon in a greenhouse in Dengkou County. As a pre-experiment, we harvested fruits at 18 DAP and 36 DAP. The mesocarp of fruit was kept, and 3 replicates were obtained from different plants for each sample. Sample processing and sequencing were the same as those of 2016. The sequencing data were already submitted to the NCBI SRA archive (accession number: PRJNA624184).

### Total RNA extraction

Total RNA was extracted from the mesocarp samples of four stages (G, R, C, and P), roots, stems, leaves and cotyledon tissues using TRIzol reagent (Invitrogen, United States) according to the manufacturer’s instructions. Total RNA concentration and purity were measured by a NanoDrop 2000 (Thermo Scientific), and total RNA integration and quality were assayed by agarose gel electrophoresis. Then, the genomic DNA was removed by RNase-free DNase I (TaKaRa, Dalian, China), and the total RNA was dissolved in RNase-free water (Tiangen, Beijing, China). The sample for degradome sequencing was composed of equal amounts of the four stage samples to obtain a sufficiently large sample.

### Small RNA libraries and degradome library construction and sequencing

Total RNA samples (G, R, C, and P) were sent to Shanghai Personal Biotechnology Co., Ltd. (China) to construct the small RNA (sRNA) libraries. Then, single-end sequencing was conducted by using an Illumina NextSeq 500 sequencing system.

To construct a cDNA library for degradome sequencing, poly(A) RNA was purified from the fruit total RNA (20 μg) using poly-T oligo-attached magnetic beads (Roche, USA) and two rounds of purification. Because the 3’ cleavage product of the mRNA contained a 5’-monophosphate, the 5’ adapters were ligated to the 5’ end of the 3’ cleavage product of the mRNA by RNA ligase. Reverse transcription was performed to make the first strand of cDNA with a 3’-adapter random primer, and size selection was performed with AMPure XP beads (Beckman, USA). Then, the cDNA was amplified with PCR under the following conditions: initial denaturation at 95 °C for 3 min; 15 cycles of denaturation at 98 °C for 15 s, annealing at 60 °C for 15 s, and extension at 72 °C for 30 s; and a final extension at 72 °C for 5 min. The average insert size for the final cDNA library was 200-400 bp. Finally, we performed 50 bp single-end sequencing on an Illumina HiSeq 2500 following the vendor’s recommended protocol. The raw data of the small RNA libraries were submitted to the NCBI SRA (Short-Read Archive) database (accession number: PRJNA624184).

### Data analysis and retrieval

To analyze the miRNA sequencing data, the raw data were first filtered to trim the sequencing adapters and remove the low-quality reads. Then, the reads with lengths between 18 nt and 36 nt were counted, and identical reads were removed to obtain unique reads. The Rfam 11 database was used to identify ribosomal RNAs (rRNAs), transfer RNAs (tRNAs), small nuclear RNAs (snRNAs) and small nucleolar RNAs (snoRNAs) by blasting the unique reads against the database^44^. The reads that were not matched to the four kinds of RNA were used for the next step of the analysis. These reads were blasted against the mature melon miRNA sequences (miRBase 21, http://www.mirbase.org/) to identify known conserved miRNAs^45^. The expression of miRNAs was normalized by read counts per million (CPM). DESeq (version 1.18.0) software was used to calculate the miRNA differential expression^46^. The cutoff value for miRNAs was a log2|fold change|> 1 and a *p*-value < 0.05. Hierarchical clustering and heatmap were performed in R by the pheatmap package (Version 1.0.12, Raivo Kolde). The Euclidean distance was calculated, and complete linkage was used for clustering. PsRobot was used to predict miRNA targets with the default setting^47^. The secondary structure of miRNA precursors was predicted by mFold (version 3.5)^48^. To identify novel miRNAs expressed in melon fruit, Mireap (https://sourceforge.net/projects/mireap) was used with the default settings, novel miRNA was named by the software based on the predicted novel miRNA candidate order, and the minimum free energy (MFE) and MFE index (MFEI) were also investigated^49^. MFEI can be easily used to distinguish miRNAs from other noncoding and coding RNAs because most miRNA precursors have an MFEI greater than 0.85. To obtain a more precise prediction of novel miRNAs, the novel miRNA annotation followed the revised criteria for plant miRNA annotation^50^. Transcriptome data (SRA accession: PRJNA543288) were obtained from the SRA database (https://www.ncbi.nlm.nih.gov/sra).

For degradome sequencing data analysis and target gene determination, the raw data were trimmed by FastQC to obtain clean reads. ACGT101-DGD (3.1), CleaveLand and Target Finder software were used for subsequent analysis^51^. Reads were mapped to melon cDNA to generate the degradome density file. The target mRNA was predicted by Target Finder software, and the result was compared to the degradome density file to determine the copredicted mRNAs. A T-plot was used to show the target mRNAs of the miRNAs^52^. The target genes of miRNA were determined by the degradome category and the *P* value of the T-plot. To obtain more rigorous results, the genes of degradome categories 0, 1, and 2 with *P* values <0.05 were retained for later miRNA target gene analysis. The mRNA target gene ontology (GO) analyses were performed by Metascape (http://metascape.org/)^53^.

### Generation of transgenic melon plant lines

The hairpin sequence of the cme-miR393a sequence (pre-miR393a) was amplified using specific primers (Supplementary Table 4), and the fragment was cloned into the pPZP221 vector under the transcriptional control of the 35S promotor. The constructs were then introduced into wild-type Hetao melon plants by pollen tube pathway^54^. Transformed plants were selected using specific primers by PCR to confirm the presence of T-DNA inserts in the transgenic lines. More than three independent lines were obtained. Transgenic lines were grown in the greenhouse as previously mentioned, and fruit phenotype observation, fruit soluble solids content and firmness were performed on the T1, T2, and T3 generations.

### Quantitative real-time PCR analysis

A quantitative real-time PCR (qRT-PCR) assay was performed to validate the miRNA expression. Total RNA was reverse transcribed using an oligo (dT) primer with a Mir-X miRNA First-Strand Synthesis kit (TaKaRa, Dalian, China). The 5’ forward primers for qRT-PCR validation of miRNAs included the entire sequence of the mature miRNAs, as suggested by the manufacturer, and the 3’ primer for qRT-PCR was supplied with the kit. U6 small nuclear RNA was used as the internal control. The primers are listed in Supplementary Table 4. qRT-PCR was performed using SYBR^®^ Premix Ex Taq™ II (TaKaRa, Dalian, China) and a 96-well Chromo4 Real-Time PCR system (Bio-Rad). The qRT-PCR conditions were as follows: predenaturation for 30 s at 95 °C, followed by 40 cycles of 5 s at 95 °C and 30 s at 60 °C.

Quantitative real-time PCR of miRNA target gene analysis was performed using SYBR® Premix Ex Taq™ II (TaKaRa, Dalian, China) and a 96-well Chromo4 Real-Time PCR system (Bio-Rad). *GAPDH* was used as an internal control gene. The qRT-PCR conditions were as follows: predenaturation for 30 s at 95 °C, followed by 40 cycles of 5 s at 95 °C and 30 s at 60 °C. The delta-delta Ct method was used to analyze the miRNA and mRNA expression levels.

## Supplementary information

Supplementary file
