# Supplementary material for: Genome-wide identification of microRNAs involved in the regulation of fruit ripening and climacteric stages in melon (Cucumis melo)
Source: Hortic Res. 2020 Jul 1;7:106. doi: 10.1038/s41438-020-0331-3 (PMC7327070; doi:10.1038/s41438-020-0331-3)
Supplement: Supplementary file 1 — Supplementary file [file 41438_2020_331_MOESM1_ESM.docx]

Supplementary Information


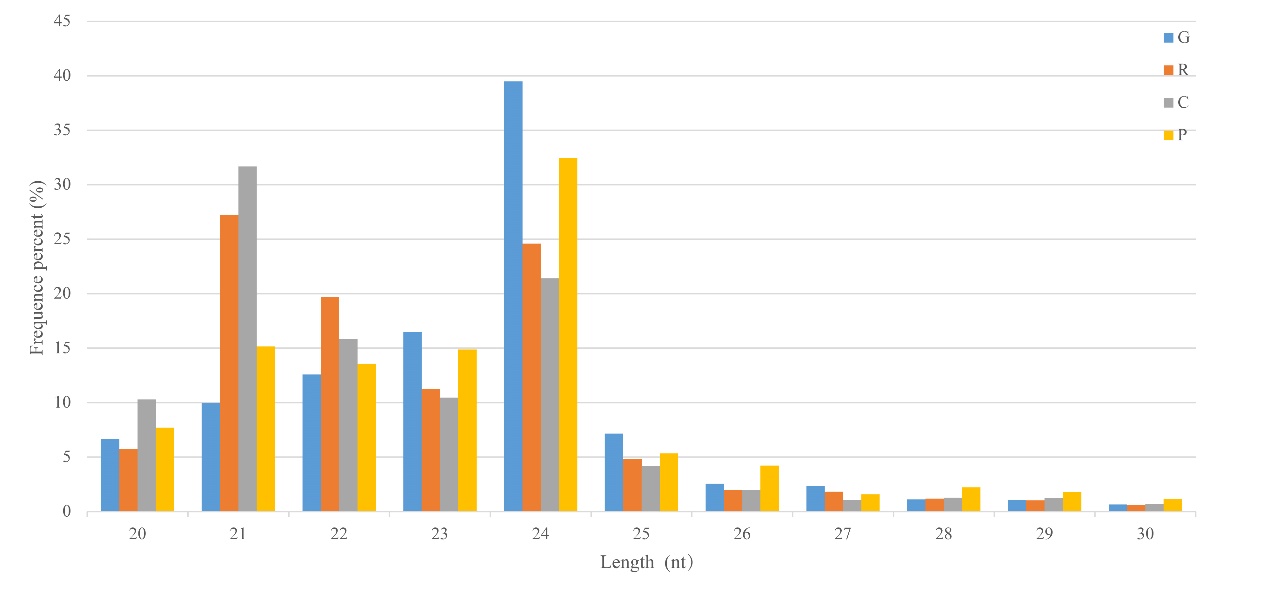
Supplement Fig. 1

**Supplementary Fig 1** The unique length distribution of sRNA. From 20 nt to 30 nt length sRNA were counted. Each bar chart stands for a statistic of the three replicate libraries.


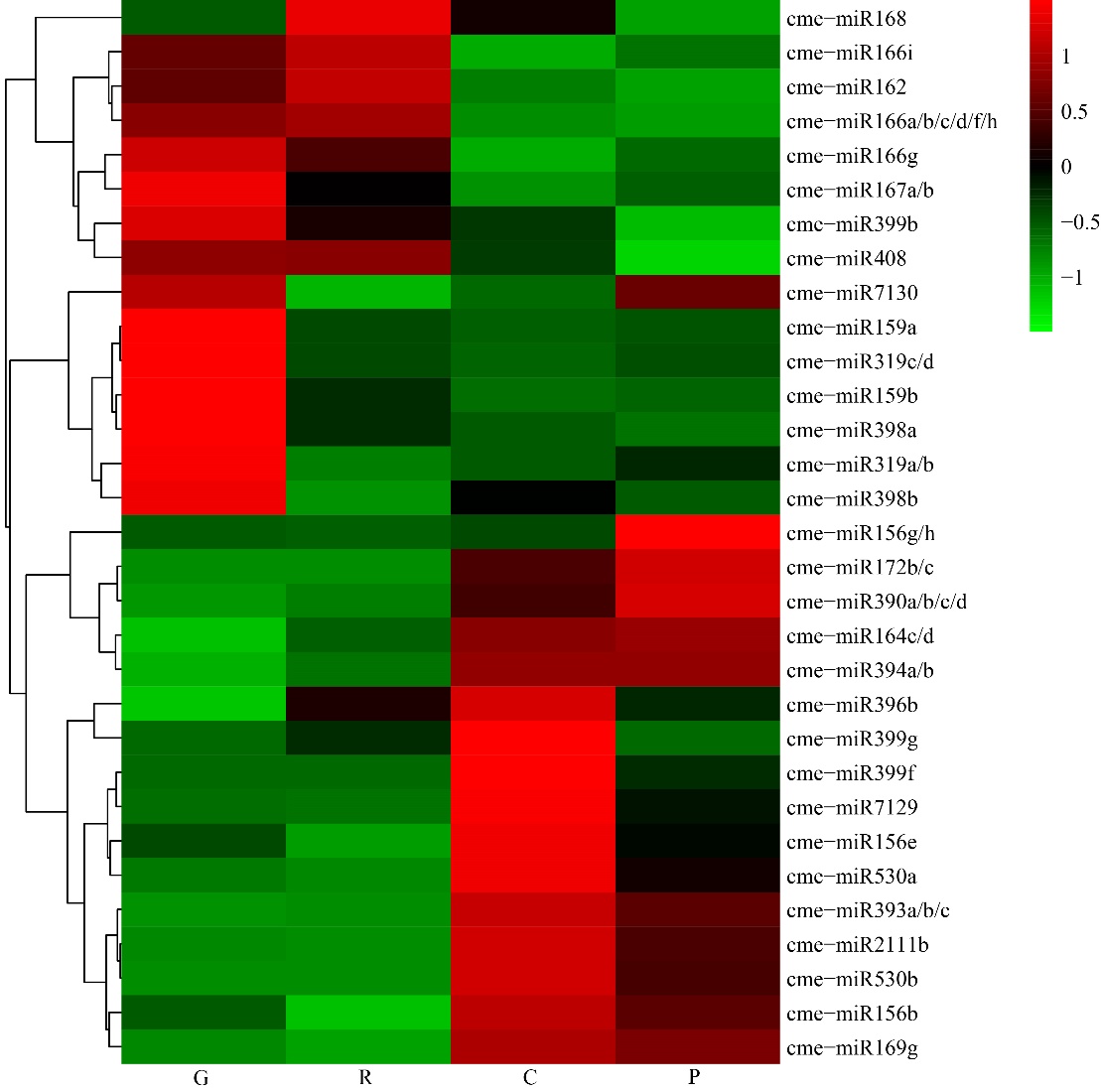
Supplement Fig. 2

**Supplementary Fig 2** Differential expression profile of conserved miRNA. Expression profile and clustering of differential expressed conserved miRNA.

**Supplementary Table 1** Total reads of miRNA sequencing.

| **Libaray** | **raw reads** | **clean reads** | **unique reads** | **Known miRNA** | **Novel miRNA candidate** | **rRNA** | **snoRNA** | **snRNA** | **tRNA** | **unknown** |
| --- | --- | --- | --- | --- | --- | --- | --- | --- | --- | --- |
| G1 | 14346417 | 11402524 | 4690615 | 98394 | 1953 | 2876954 | 43887 | 27983 | 569493 | 7783860 |
| G2 | 14825465 | 9173943 | 3392091 | 95589 | 2290 | 2677701 | 34929 | 21765 | 597584 | 5744085 |
| G3 | 14946088 | 11843365 | 4551419 | 113302 | 3282 | 2714760 | 47892 | 26198 | 550110 | 8387821 |
| R1 | 12493036 | 10666318 | 2852972 | 36623 | 982 | 1205532 | 68797 | 43084 | 228471 | 9082829 |
| R2 | 12603361 | 11495995 | 3647131 | 50805 | 1408 | 1412998 | 63959 | 42971 | 306339 | 9617515 |
| R3 | 12313964 | 10390401 | 3777020 | 43445 | 1889 | 2479117 | 44698 | 27027 | 578031 | 7216194 |
| C1 | 13223598 | 7050998 | 2656791 | 38923 | 646 | 1687719 | 61280 | 33986 | 134158 | 5094286 |
| C2 | 13238691 | 10431933 | 3460563 | 48106 | 1468 | 1458669 | 81826 | 51087 | 157340 | 8633437 |
| C3 | 12271940 | 9438359 | 2400235 | 23049 | 753 | 1662477 | 78480 | 34448 | 396602 | 7242550 |
| P1 | 11332673 | 8971208 | 3189926 | 29437 | 775 | 1825308 | 70447 | 27285 | 642558 | 6375398 |
| P2 | 13541175 | 11097547 | 4496258 | 39806 | 1237 | 2099248 | 68839 | 35017 | 290296 | 8563104 |
| P3 | 12914014 | 10956233 | 4465153 | 46203 | 1279 | 1702679 | 66111 | 34905 | 222151 | 8882905 |

**Supplementary Table 2 Conserved miRNAs in Hetao melon**

| **Family** | **miRNA** | **G1** | **G2** | **G3** | **R1** | **R2** | **R3** | **C1** | **C2** | **C3** | **P1** | **P2** | **P3** |
| --- | --- | --- | --- | --- | --- | --- | --- | --- | --- | --- | --- | --- | --- |
| miR156 | cme-miR156b | 1.40 | 2.29 | 3.04 | 0.94 | 1.39 | 1.25 | 9.22 | 3.36 | 2.75 | 1.89 | 6.76 | 3.65 |
|  | cme-miR156e | 0.35 | 0.11 | 0.17 | 0.00 | 0.00 | 0.19 | 0.99 | 0.86 | 0.42 | 0.33 | 0.54 | 0.09 |
|  | cme-miR156f | 0.26 | 0.00 | 0.08 | 0.00 | 0.00 | 0.00 | 0.43 | 0.19 | 0.32 | 0.33 | 0.99 | 0.27 |
|  | cme-miR156g/h | 0.09 | 0.44 | 0.25 | 0.00 | 0.35 | 0.38 | 0.85 | 0.10 | 0.11 | 2.12 | 2.16 | 1.92 |
|  | cme-miR156j | 53.41 | 81.10 | 91.02 | 39.47 | 37.40 | 111.45 | 124.38 | 34.41 | 44.92 | 128.63 | 67.49 | 34.96 |
| miR159 | cme-miR159a | 5611.39 | 7706.61 | 6174.93 | 1217.29 | 1214.68 | 1935.92 | 1608.71 | 1315.86 | 590.57 | 1192.37 | 1343.27 | 1424.03 |
|  | cme-miR159b | 455.78 | 521.59 | 420.57 | 87.85 | 101.08 | 137.34 | 53.04 | 15.43 | 8.26 | 37.79 | 38.30 | 48.10 |
|  | cme-miR319a/b | 122.78 | 198.39 | 417.11 | 29.25 | 31.14 | 33.01 | 76.58 | 44.38 | 29.98 | 47.71 | 54.79 | 139.28 |
|  | cme-miR319c/d | 114.01 | 149.45 | 280.83 | 42.75 | 35.75 | 40.61 | 50.49 | 23.49 | 14.20 | 29.20 | 26.76 | 57.32 |
| miR160 | cme-miR160a/b/c | 0.44 | 0.55 | 0.59 | 0.75 | 0.26 | 0.10 | 0.85 | 0.00 | 0.21 | 0.33 | 0.27 | 0.00 |
| miR162_1 | cme-miR162 | 257.93 | 219.53 | 308.78 | 343.14 | 450.07 | 183.53 | 179.83 | 69.50 | 79.15 | 84.38 | 98.49 | 78.13 |
| miR164 | cme-miR164a | 8.24 | 4.25 | 11.82 | 2.16 | 2.26 | 5.20 | 5.39 | 3.16 | 2.33 | 3.12 | 2.97 | 5.11 |
|  | cme-miR164b | 0.79 | 0.11 | 0.76 | 0.00 | 0.09 | 0.00 | 0.28 | 0.38 | 0.00 | 0.22 | 0.09 | 0.27 |
|  | cme-miR164c/d | 105.50 | 107.48 | 92.88 | 162.10 | 193.11 | 246.86 | 573.82 | 524.73 | 188.80 | 395.93 | 376.30 | 558.22 |
| miR166 | cme-miR166a/b/c/d/f/h | 239.60 | 138.22 | 216.15 | 211.79 | 174.06 | 239.26 | 119.27 | 92.31 | 55.41 | 91.96 | 75.69 | 81.87 |
|  | cme-miR166e | 209.25 | 132.44 | 205.09 | 171.38 | 197.98 | 127.14 | 186.21 | 139.48 | 99.91 | 167.54 | 179.59 | 164.56 |
|  | cme-miR166g | 223.63 | 114.45 | 169.21 | 110.16 | 124.74 | 148.41 | 55.03 | 68.54 | 30.09 | 79.70 | 70.83 | 68.45 |
|  | cme-miR166i | 71.21 | 45.67 | 71.52 | 84.85 | 91.68 | 57.17 | 25.53 | 8.44 | 4.45 | 31.21 | 18.56 | 20.26 |
| miR167_1 | cme-miR167a/b | 7.98 | 4.25 | 5.23 | 2.25 | 3.57 | 2.89 | 2.69 | 0.67 | 0.00 | 1.78 | 1.89 | 1.46 |
|  | cme-miR167c | 5.79 | 1.96 | 2.79 | 2.72 | 2.35 | 1.35 | 3.40 | 1.05 | 1.27 | 3.90 | 2.88 | 4.38 |
|  | cme-miR167d/f | 40.69 | 20.17 | 25.92 | 9.38 | 13.74 | 11.55 | 21.13 | 14.86 | 8.16 | 20.73 | 19.73 | 21.36 |
| miR168 | cme-miR168 | 33.50 | 22.67 | 34.37 | 115.69 | 134.05 | 17.03 | 120.12 | 20.03 | 9.43 | 19.84 | 14.51 | 18.89 |
| miR169 | cme-miR169t | 2.98 | 1.96 | 1.18 | 1.13 | 2.70 | 2.31 | 4.40 | 15.53 | 3.07 | 3.57 | 8.20 | 10.13 |
| miR169_1 | cme-miR169c/d/o | 1.58 | 0.11 | 0.00 | 1.13 | 1.74 | 1.15 | 1.84 | 4.12 | 0.85 | 1.23 | 2.61 | 1.10 |
|  | cme-miR169i/j/l/m | 3.16 | 0.87 | 1.35 | 0.94 | 2.70 | 0.87 | 0.43 | 2.49 | 0.21 | 1.78 | 4.51 | 3.10 |
| miR169_2 | cme-miR169g | 0.09 | 0.11 | 0.17 | 0.00 | 0.09 | 0.00 | 1.56 | 1.73 | 0.32 | 0.22 | 2.25 | 0.55 |
|  | cme-miR169k | 0.00 | 0.00 | 0.00 | 0.00 | 0.00 | 0.00 | 0.28 | 0.10 | 0.00 | 0.00 | 0.27 | 0.55 |
| miR169_8 | cme-miR169p/q/s | 0.00 | 0.00 | 0.00 | 0.00 | 0.17 | 0.10 | 0.14 | 0.00 | 0.00 | 0.00 | 0.09 | 0.18 |
| miR171_1 | cme-miR171f | 0.00 | 0.00 | 0.00 | 0.09 | 0.17 | 0.00 | 0.14 | 0.10 | 0.00 | 0.33 | 0.18 | 0.37 |
| miR172 | cme-miR172a | 0.00 | 0.11 | 0.08 | 0.00 | 0.09 | 0.10 | 0.57 | 0.10 | 0.32 | 0.78 | 0.72 | 0.73 |
|  | cme-miR172b/c | 0.00 | 0.00 | 0.00 | 0.00 | 0.00 | 0.00 | 0.43 | 0.48 | 0.21 | 0.33 | 0.18 | 1.28 |
|  | cme-miR172d | 0.09 | 0.22 | 0.00 | 0.00 | 0.00 | 0.00 | 0.14 | 0.10 | 0.00 | 0.00 | 0.09 | 0.00 |
|  | cme-miR172e | 0.00 | 0.00 | 0.00 | 0.19 | 0.00 | 0.00 | 0.14 | 0.38 | 0.32 | 0.11 | 0.27 | 0.27 |
| miR1863 | cme-miR1863 | 1.05 | 0.65 | 0.84 | 0.84 | 0.52 | 0.38 | 0.99 | 0.77 | 0.53 | 1.00 | 0.54 | 0.82 |
| miR2111 | cme-miR2111b | 2.19 | 0.11 | 0.68 | 0.28 | 0.35 | 0.19 | 36.59 | 95.28 | 7.52 | 21.74 | 20.27 | 44.63 |
| miR390 | cme-miR390a/b/c/d | 0.26 | 0.55 | 0.76 | 0.38 | 1.13 | 3.46 | 6.52 | 17.06 | 8.37 | 13.26 | 15.05 | 25.19 |
| miR393 | cme-miR393a/b/c | 0.26 | 0.87 | 0.51 | 0.38 | 2.61 | 0.87 | 62.26 | 20.51 | 14.41 | 13.04 | 33.70 | 19.99 |
| miR394 | cme-miR394a/b | 0.09 | 0.55 | 0.76 | 0.56 | 2.44 | 0.77 | 9.93 | 3.07 | 1.17 | 7.58 | 2.70 | 3.74 |
| miR395 | cme-miR395a/d/e/f | 7.98 | 21.47 | 16.97 | 7.88 | 4.87 | 4.33 | 2.55 | 3.26 | 0.95 | 0.33 | 2.79 | 1.64 |
|  | cme-miR395b/c | 11.05 | 19.84 | 11.06 | 3.38 | 6.96 | 3.66 | 2.84 | 1.25 | 1.59 | 0.56 | 5.14 | 2.28 |
| miR396 | cme-miR396a/c/d | 532.43 | 383.91 | 424.96 | 281.82 | 670.15 | 364.76 | 514.54 | 215.20 | 162.10 | 236.98 | 329.35 | 483.10 |
|  | cme-miR396b | 165.14 | 70.96 | 70.84 | 186.57 | 469.90 | 90.08 | 374.84 | 605.16 | 132.44 | 131.31 | 202.48 | 282.03 |
|  | cme-miR396e | 4.56 | 3.05 | 2.53 | 4.78 | 5.48 | 3.08 | 5.67 | 3.83 | 0.85 | 1.34 | 1.80 | 3.74 |
| miR397 | cme-miR397 | 1.40 | 1.31 | 2.53 | 0.47 | 0.43 | 2.12 | 1.13 | 1.25 | 0.53 | 0.45 | 0.99 | 0.37 |
| miR398 | cme-miR398a | 24.21 | 28.78 | 24.57 | 6.00 | 6.00 | 3.66 | 2.41 | 2.68 | 0.64 | 0.00 | 0.09 | 0.55 |
|  | cme-miR398b | 1.84 | 0.65 | 0.68 | 0.28 | 0.00 | 0.00 | 0.43 | 0.38 | 0.53 | 0.00 | 0.45 | 0.27 |
| miR399 | cme-miR399a | 1.49 | 0.65 | 1.35 | 0.19 | 0.09 | 0.48 | 0.00 | 0.10 | 0.00 | 0.00 | 0.00 | 0.00 |
|  | cme-miR399b | 12.89 | 24.63 | 31.24 | 7.97 | 8.26 | 20.40 | 1.99 | 11.50 | 10.28 | 0.11 | 0.63 | 1.00 |
|  | cme-miR399c | 0.96 | 0.11 | 0.42 | 0.47 | 0.26 | 0.19 | 0.00 | 1.25 | 0.74 | 0.11 | 0.27 | 0.09 |
|  | cme-miR399d/e | 4.65 | 8.83 | 8.19 | 1.78 | 3.48 | 7.51 | 4.82 | 11.69 | 11.44 | 2.12 | 2.25 | 5.84 |
|  | cme-miR399f | 0.00 | 0.00 | 0.00 | 0.00 | 0.00 | 0.00 | 0.28 | 1.34 | 0.95 | 0.00 | 0.09 | 0.37 |
|  | cme-miR399g | 47.36 | 48.62 | 66.54 | 30.94 | 122.56 | 144.17 | 107.64 | 426.29 | 467.88 | 33.22 | 57.58 | 70.64 |
| miR408 | cme-miR408 | 9.21 | 10.46 | 9.46 | 6.94 | 11.74 | 9.91 | 2.69 | 9.78 | 1.70 | 0.89 | 1.08 | 0.64 |
| miR477 | cme-miR477b | 26.75 | 37.61 | 61.72 | 23.63 | 54.54 | 28.58 | 18.86 | 43.42 | 12.29 | 31.77 | 60.55 | 48.56 |
| miR530 | cme-miR530a | 0.09 | 0.00 | 0.00 | 0.00 | 0.00 | 0.00 | 1.28 | 0.38 | 0.42 | 0.56 | 0.27 | 0.00 |
|  | cme-miR530b | 0.96 | 0.22 | 0.17 | 1.03 | 2.78 | 1.15 | 235.14 | 245.88 | 68.02 | 166.42 | 98.04 | 68.91 |
| miR7129 | cme-miR7129 | 159.96 | 227.17 | 224.26 | 216.76 | 211.90 | 163.04 | 873.21 | 453.99 | 340.10 | 241.10 | 280.51 | 356.24 |
| miR7130 | cme-miR7130 | 24.73 | 42.18 | 57.75 | 10.78 | 10.53 | 16.75 | 17.73 | 25.40 | 12.71 | 24.08 | 39.92 | 42.53 |
| miR845 | cme-miR845 | 0.09 | 0.22 | 0.08 | 0.00 | 0.09 | 0.29 | 0.57 | 0.38 | 0.42 | 0.45 | 1.08 | 0.18 |
| miR854 | cme-miR854 | 0.00 | 0.11 | 0.08 | 0.00 | 0.17 | 0.00 | 0.00 | 0.00 | 0.00 | 0.00 | 0.18 | 0.00 |
| miR858 | cme-miR858 | 15.52 | 11.01 | 11.82 | 2.06 | 4.61 | 6.16 | 6.52 | 3.93 | 7.42 | 3.46 | 6.85 | 2.65 |

Note: G: growth stage sample; R: ripening stage sample; C: climacteric stage sample; P: post-climacteric stage sample.

**Supplementary Table 3** Table of Target genes of degradome sequencing.

| **SmallRNA** | **Transcript** | **Gene** | **Transcript**  **Annotation** | **Alignment**  **Score** | **Alignment**  **Range** | **Degradome**  **CleavageSite** | **Degradome**  **Category** | **Degradome**  **Pvalue** |
| --- | --- | --- | --- | --- | --- | --- | --- | --- |
| cme-miR156b | MELO3C005966T1 | MELO3C005966 | uncharacterized LOC103401427 | 3 | 833-853 | 844 | 2 | 0.803 |
| cme-miR156b | MELO3C009639T1 | MELO3C009639 | - | 2 | 1026-1046 | 1037 | 2 | 0.734 |
| cme-miR156b | MELO3C009639T3 | MELO3C009639 | - | 2 | 287-307 | 298 | 2 | 0.734 |
| cme-miR156e | MELO3C005966T1 | MELO3C005966 | uncharacterized LOC103401427 | 3.5 | 833-853 | 844 | 2 | 0.764 |
| cme-miR156e | MELO3C009639T1 | MELO3C009639 | - | 2.5 | 1026-1046 | 1037 | 2 | 0.7 |
| cme-miR156e | MELO3C009639T3 | MELO3C009639 | - | 2.5 | 287-307 | 298 | 2 | 0.7 |
| cme-miR156f | MELO3C005966T1 | MELO3C005966 | uncharacterized LOC103401427 | 2 | 833-852 | 843 | 0 | 0.0365 |
| cme-miR156f | MELO3C009639T1 | MELO3C009639 | - | 2 | 1026-1045 | 1036 | 2 | 0.949 |
| cme-miR156f | MELO3C026197T1 | MELO3C026197 | - | 2 | 362-381 | 372 | 0 | 0.0365 |
| cme-miR156f | MELO3C009639T3 | MELO3C009639 | - | 2 | 287-306 | 297 | 0 | 0.0365 |
| cme-miR156g | MELO3C005966T1 | MELO3C005966 | uncharacterized LOC103401427 | 0.5 | 833-852 | 843 | 0 | 0.0462 |
| cme-miR156g | MELO3C009639T1 | MELO3C009639 | - | 0.5 | 1026-1045 | 1036 | 2 | 0.977 |
| cme-miR156g | MELO3C014678T1 | MELO3C014678 | - | 1.5 | 531-549 | 540 | 2 | 0.661 |
| cme-miR156g | MELO3C026197T1 | MELO3C026197 | - | 0.5 | 362-381 | 372 | 0 | 0.0462 |
| cme-miR156g | MELO3C009639T3 | MELO3C009639 | - | 0.5 | 287-306 | 297 | 0 | 0.0462 |
| cme-miR159a | MELO3C016121T1 | MELO3C016121 | BES1/BZR1 homolog protein 2 | 3 | 309-329 | 320 | 1 | 0.0124 |
| cme-miR159a | MELO3C025628T1 | MELO3C025628 | WAS/WASL-interacting protein family member 1-like | 3 | 945-965 | 956 | 0 | 0.0101 |
| cme-miR159a | MELO3C016121T2 | MELO3C016121 | BES1/BZR1 homolog protein 2 | 3 | 897-917 | 908 | 2 | 0.556 |
| cme-miR159a | MELO3C023869T2 | MELO3C023869 | - | 4 | 2072-2092 | 2083 | 2 | 0.814 |
| cme-miR159a | MELO3C023869T1 | MELO3C023869 | - | 4 | 2075-2095 | 2086 | 2 | 0.814 |
| cme-miR159a | MELO3C019923T1 | MELO3C019923 | myb domain protein 30 | 3.5 | 941-961 | 952 | 0 | 0.00599 |
| cme-miR159b | MELO3C019923T1 | MELO3C019923 | myb domain protein 30 | 3 | 941-961 | 952 | 0 | 0.00673 |
| cme-miR160c | MELO3C019801T1 | MELO3C019801 | auxin response factor 7-like | 1 | 1335-1355 | 1346 | 0 | 0.00971 |
| cme-miR160c | MELO3C011372T1 | MELO3C011372 | auxin response factor 7-like | 0.5 | 1457-1477 | 1468 | 0 | 0.0105 |
| cme-miR160c | MELO3C014232T1 | MELO3C014232 | auxin response factor 7-like | 0.5 | 1351-1371 | 1362 | 0 | 0.0105 |
| cme-miR160c | MELO3C025777T1 | MELO3C025777 | auxin response factor 7-like | 1 | 1762-1782 | 1773 | 0 | 0.00971 |
| cme-miR160d | MELO3C019801T1 | MELO3C019801 | auxin response factor 7-like | 2 | 1335-1355 | 1346 | 0 | 0.00822 |
| cme-miR160d | MELO3C011372T1 | MELO3C011372 | auxin response factor 7-like | 1 | 1457-1477 | 1468 | 0 | 0.00971 |
| cme-miR160d | MELO3C014232T1 | MELO3C014232 | auxin response factor 7-like | 1 | 1351-1371 | 1362 | 0 | 0.00971 |
| cme-miR160d | MELO3C025777T1 | MELO3C025777 | auxin response factor 7-like | 2 | 1762-1782 | 1773 | 0 | 0.00822 |
| cme-miR162 | MELO3C005929T1 | MELO3C005929 | ribonuclease III | 2 | 298-319 | 309 | 2 | 0.282 |
| cme-miR164a | MELO3C010555T1 | MELO3C010555 | NAC domain-containing protein 89-like | 1 | 620-640 | 631 | 0 | 0.00487 |
| cme-miR164a | MELO3C001996T1 | MELO3C001996 | NAC domain-containing protein 89-like | 2 | 705-725 | 716 | 0 | 0.0123 |
| cme-miR164a | MELO3C017185T1 | MELO3C017185 | NAC domain-containing protein 89-like | 2 | 659-679 | 670 | 0 | 0.0123 |
| cme-miR164a | MELO3C022002T1 | MELO3C022002 | NAC domain-containing protein 89-like | 3 | 644-664 | 655 | 0 | 0.00673 |
| cme-miR164b | MELO3C010555T1 | MELO3C010555 | NAC domain-containing protein 89-like | 2 | 620-640 | 631 | 0 | 0.00412 |
| cme-miR164b | MELO3C001996T1 | MELO3C001996 | NAC domain-containing protein 89-like | 4 | 705-725 | 716 | 0 | 0.00785 |
| cme-miR164b | MELO3C017185T1 | MELO3C017185 | NAC domain-containing protein 89-like | 4 | 659-679 | 670 | 0 | 0.00785 |
| cme-miR164d | MELO3C010555T1 | MELO3C010555 | NAC domain-containing protein 89-like | 2 | 620-640 | 631 | 0 | 0.0123 |
| cme-miR164d | MELO3C001996T1 | MELO3C001996 | NAC domain-containing protein 89-like | 2 | 705-725 | 716 | 0 | 0.0123 |
| cme-miR164d | MELO3C022002T1 | MELO3C022002 | NAC domain-containing protein 89-like | 3 | 644-664 | 655 | 0 | 0.0134 |
| cme-miR164d | MELO3C017185T1 | MELO3C017185 | NAC domain-containing protein 89-like | 3 | 659-679 | 670 | 0 | 0.0134 |
| cme-miR166a | MELO3C024446T2 | MELO3C024446 | - | 2.5 | 610-630 | 621 | 2 | 0.452 |
| cme-miR166a | MELO3C025774T1 | MELO3C025774 | - | 3 | 574-594 | 585 | 0 | 0.0299 |
| cme-miR166a | MELO3C025774T2 | MELO3C025774 | - | 3 | 574-594 | 585 | 0 | 0.0299 |
| cme-miR166a | MELO3C025774T4 | MELO3C025774 | - | 3 | 574-594 | 585 | 0 | 0.0299 |
| cme-miR166a | MELO3C025774T3 | MELO3C025774 | - | 3 | 574-594 | 585 | 0 | 0.0299 |
| cme-miR166a | MELO3C003468T1 | MELO3C003468 | - | 3 | 973-993 | 984 | 2 | 0.912 |
| cme-miR166a | MELO3C002807T1 | MELO3C002807 | - | 3 | 1379-1399 | 1390 | 1 | 0.0368 |
| cme-miR166a | MELO3C024446T1 | MELO3C024446 | - | 2.5 | 551-571 | 562 | 0 | 0.00748 |
| cme-miR166a | MELO3C004887T1 | MELO3C004887 | - | 3 | 563-583 | 574 | 0 | 0.0299 |
| cme-miR166a | MELO3C007078T2 | MELO3C007078 | - | 3 | 569-589 | 580 | 0 | 0.0299 |
| cme-miR166a | MELO3C007078T1 | MELO3C007078 | - | 3 | 569-589 | 580 | 0 | 0.0299 |
| cme-miR166e | MELO3C024446T2 | MELO3C024446 | - | 2.5 | 610-630 | 621 | 2 | 0.452 |
| cme-miR166e | MELO3C025774T1 | MELO3C025774 | - | 3 | 574-594 | 585 | 0 | 0.0299 |
| cme-miR166e | MELO3C025774T2 | MELO3C025774 | - | 3 | 574-594 | 585 | 0 | 0.0299 |
| cme-miR166e | MELO3C025774T4 | MELO3C025774 | - | 3 | 574-594 | 585 | 0 | 0.0299 |
| cme-miR166e | MELO3C025774T3 | MELO3C025774 | - | 3 | 574-594 | 585 | 0 | 0.0299 |
| cme-miR166e | MELO3C003468T1 | MELO3C003468 | - | 3 | 973-993 | 984 | 2 | 0.912 |
| cme-miR166e | MELO3C002807T1 | MELO3C002807 | - | 3 | 1379-1399 | 1390 | 1 | 0.0368 |
| cme-miR166e | MELO3C024446T1 | MELO3C024446 | - | 2.5 | 551-571 | 562 | 0 | 0.00748 |
| cme-miR166e | MELO3C004887T1 | MELO3C004887 | - | 3 | 563-583 | 574 | 0 | 0.0299 |
| cme-miR166e | MELO3C007078T2 | MELO3C007078 | - | 3 | 569-589 | 580 | 0 | 0.0299 |
| cme-miR166e | MELO3C007078T1 | MELO3C007078 | - | 3 | 569-589 | 580 | 0 | 0.0299 |
| cme-miR166g | MELO3C024446T2 | MELO3C024446 | - | 1.5 | 611-630 | 621 | 2 | 0.514 |
| cme-miR166g | MELO3C025774T1 | MELO3C025774 | - | 2 | 575-594 | 585 | 0 | 0.0365 |
| cme-miR166g | MELO3C025774T2 | MELO3C025774 | - | 2 | 575-594 | 585 | 0 | 0.0365 |
| cme-miR166g | MELO3C025774T4 | MELO3C025774 | - | 2 | 575-594 | 585 | 0 | 0.0365 |
| cme-miR166g | MELO3C025774T3 | MELO3C025774 | - | 2 | 575-594 | 585 | 0 | 0.0365 |
| cme-miR166g | MELO3C003468T1 | MELO3C003468 | - | 2 | 974-993 | 984 | 2 | 0.949 |
| cme-miR166g | MELO3C002807T1 | MELO3C002807 | - | 2 | 1380-1399 | 1390 | 1 | 0.0448 |
| cme-miR166g | MELO3C024446T1 | MELO3C024446 | - | 1.5 | 552-571 | 562 | 0 | 0.00897 |
| cme-miR166g | MELO3C004887T1 | MELO3C004887 | - | 2 | 564-583 | 574 | 0 | 0.0365 |
| cme-miR166g | MELO3C007078T2 | MELO3C007078 | - | 2 | 570-589 | 580 | 0 | 0.0365 |
| cme-miR166g | MELO3C007078T1 | MELO3C007078 | - | 2 | 570-589 | 580 | 0 | 0.0365 |
| cme-miR166i | MELO3C024446T2 | MELO3C024446 | - | 2 | 611-630 | 621 | 2 | 0.484 |
| cme-miR166i | MELO3C025774T1 | MELO3C025774 | - | 2.5 | 575-594 | 585 | 0 | 0.0332 |
| cme-miR166i | MELO3C025774T2 | MELO3C025774 | - | 2.5 | 575-594 | 585 | 0 | 0.0332 |
| cme-miR166i | MELO3C025774T4 | MELO3C025774 | - | 2.5 | 575-594 | 585 | 0 | 0.0332 |
| cme-miR166i | MELO3C025774T3 | MELO3C025774 | - | 2.5 | 575-594 | 585 | 0 | 0.0332 |
| cme-miR166i | MELO3C003468T1 | MELO3C003468 | - | 2.5 | 974-993 | 984 | 2 | 0.933 |
| cme-miR166i | MELO3C002807T1 | MELO3C002807 | - | 2.5 | 1380-1399 | 1390 | 1 | 0.0408 |
| cme-miR166i | MELO3C024446T1 | MELO3C024446 | - | 2 | 552-571 | 562 | 0 | 0.00822 |
| cme-miR166i | MELO3C004887T1 | MELO3C004887 | - | 2.5 | 564-583 | 574 | 0 | 0.0332 |
| cme-miR166i | MELO3C007078T2 | MELO3C007078 | - | 2.5 | 570-589 | 580 | 0 | 0.0332 |
| cme-miR166i | MELO3C007078T1 | MELO3C007078 | - | 2.5 | 570-589 | 580 | 0 | 0.0332 |
| cme-miR167b | MELO3C025070T1 | MELO3C025070 | auxin response factor 5-like | 4 | 2313-2334 | 2324 | 0 | 0.013 |
| cme-miR167b | MELO3C025070T2 | MELO3C025070 | auxin response factor 5-like | 4 | 1782-1803 | 1793 | 0 | 0.013 |
| cme-miR167e | MELO3C025070T1 | MELO3C025070 | auxin response factor 5-like | 2 | 2313-2333 | 2324 | 0 | 0.00822 |
| cme-miR167e | MELO3C002771T1 | MELO3C002771 | auxin response factor 5-like | 3 | 3385-3405 | 3396 | 2 | 0.418 |
| cme-miR167e | MELO3C007105T1 | MELO3C007105 | auxin response factor 5-like | 3 | 765-785 | 776 | 0 | 0.00673 |
| cme-miR167e | MELO3C025070T2 | MELO3C025070 | auxin response factor 5-like | 2 | 1782-1802 | 1793 | 0 | 0.00822 |
| cme-miR168 | MELO3C001957T1 | MELO3C001957 | - | 3 | 606-625 | 616 | 0 | 0.00337 |
| cme-miR169c | MELO3C024190T1 | MELO3C024190 | acetyl-CoA acetyltransferase | 4 | 469-488 | 479 | 2 | 0.468 |
| cme-miR169g | MELO3C014590T1 | MELO3C014590 | nuclear transcription factor Y subunit A-3 | 3 | 1210-1230 | 1221 | 0 | 0.0101 |
| cme-miR169g | MELO3C023554T1 | MELO3C023554 | nuclear transcription factor Y subunit A-3 | 2 | 1330-1350 | 1341 | 0 | 0.00822 |
| cme-miR169g | MELO3C023554T2 | MELO3C023554 | nuclear transcription factor Y subunit A-3 | 2 | 1345-1365 | 1356 | 0 | 0.00822 |
| cme-miR169g | MELO3C014590T2 | MELO3C014590 | nuclear transcription factor Y subunit A-3 | 3 | 1267-1287 | 1278 | 2 | 0.556 |
| cme-miR171f | MELO3C021146T1 | MELO3C021146 | DELLA protein GAI1 | 1 | 1538-1558 | 1549 | 0 | 0.0145 |
| cme-miR171f | MELO3C021146T2 | MELO3C021146 | DELLA protein GAI1 | 1 | 1638-1658 | 1649 | 0 | 0.0145 |
| cme-miR171f | MELO3C013947T1 | MELO3C013947 | hypothetical protein | 1 | 1281-1301 | 1292 | 2 | 0.69 |
| cme-miR171f | MELO3C017548T1 | MELO3C017548 | DELLA protein GAI1-like | 2 | 570-590 | 581 | 2 | 0.282 |
| cme-miR172a | MELO3C007572T2 | MELO3C007572 | ethylene-responsive transcription factor 1B | 2 | 1991-2011 | 2002 | 2 | 0.484 |
| cme-miR172a | MELO3C007572T1 | MELO3C007572 | ethylene-responsive transcription factor 1B | 2 | 2086-2106 | 2097 | 2 | 0.484 |
| cme-miR172c | MELO3C007572T2 | MELO3C007572 | ethylene-responsive transcription factor 1B | 3 | 1991-2011 | 2002 | 2 | 0.742 |
| cme-miR172c | MELO3C007572T1 | MELO3C007572 | ethylene-responsive transcription factor 1B | 3 | 2086-2106 | 2097 | 2 | 0.742 |
| cme-miR172d | MELO3C007572T2 | MELO3C007572 | ethylene-responsive transcription factor 1B | 3 | 1991-2011 | 2002 | 2 | 0.661 |
| cme-miR172d | MELO3C007572T1 | MELO3C007572 | ethylene-responsive transcription factor 1B | 3 | 2086-2106 | 2097 | 2 | 0.661 |
| cme-miR172e | MELO3C007572T2 | MELO3C007572 | ethylene-responsive transcription factor 1B | 2 | 1991-2011 | 2002 | 2 | 0.809 |
| cme-miR172e | MELO3C007572T1 | MELO3C007572 | ethylene-responsive transcription factor 1B | 2 | 2086-2106 | 2097 | 2 | 0.809 |
| cme-miR172f | MELO3C007572T2 | MELO3C007572 | ethylene-responsive transcription factor 1B | 3.5 | 1991-2011 | 2002 | 2 | 0.618 |
| cme-miR172f | MELO3C007572T1 | MELO3C007572 | ethylene-responsive transcription factor 1B | 3.5 | 2086-2106 | 2097 | 2 | 0.618 |
| cme-miR2111b | MELO3C014876T2 | MELO3C014876 | - | 1 | 743-763 | 754 | 0 | 0.00487 |
| cme-miR319a | MELO3C005595T4 | MELO3C005595 | - | 3 | 416-435 | 426 | 2 | 0.803 |
| cme-miR319a | MELO3C005595T3 | MELO3C005595 | - | 3 | 210-229 | 220 | 2 | 0.803 |
| cme-miR319a | MELO3C005595T5 | MELO3C005595 | - | 3 | 278-297 | 288 | 2 | 0.803 |
| cme-miR319a | MELO3C007121T1 | MELO3C007121 | - | 3.5 | 1797-1816 | 1807 | 0 | 0.00897 |
| cme-miR319a | MELO3C005595T1 | MELO3C005595 | - | 3 | 297-316 | 307 | 2 | 0.803 |
| cme-miR319a | MELO3C005595T2 | MELO3C005595 | - | 3 | 709-728 | 719 | 2 | 0.803 |
| cme-miR319a | MELO3C002754T1 | MELO3C002754 | - | 3.5 | 1483-1502 | 1493 | 0 | 0.00897 |
| cme-miR319a | MELO3C007121T2 | MELO3C007121 | - | 3.5 | 1920-1939 | 1930 | 0 | 0.00897 |
| cme-miR319a | MELO3C016092T1 | MELO3C016092 | - | 4 | 2086-2105 | 2096 | 0 | 0.0156 |
| cme-miR319a | MELO3C019923T1 | MELO3C019923 | myb domain protein 30 | 3 | 941-960 | 951 | 2 | 0.803 |
| cme-miR319d | MELO3C007121T1 | MELO3C007121 | - | 2.5 | 1797-1816 | 1807 | 0 | 0.0149 |
| cme-miR319d | MELO3C002754T1 | MELO3C002754 | - | 2.5 | 1483-1502 | 1493 | 0 | 0.0149 |
| cme-miR319d | MELO3C007121T2 | MELO3C007121 | - | 2.5 | 1920-1939 | 1930 | 0 | 0.0149 |
| cme-miR319d | MELO3C016092T1 | MELO3C016092 | - | 3 | 2086-2105 | 2096 | 0 | 0.0101 |
| cme-miR319d | MELO3C019923T1 | MELO3C019923 | myb domain protein 30 | 3 | 941-960 | 951 | 2 | 0.556 |
| cme-miR393c | MELO3C015898T1 | MELO3C015898 | TRANSPORT INHIBITOR RESPONSE 1 | 1 | 1745-1764 | 1755 | 0 | 0.00487 |
| cme-miR393c | MELO3C014799T1 | MELO3C014799 | TRANSPORT INHIBITOR RESPONSE 1-like | 2 | 2220-2240 | 2231 | 0 | 0.00412 |
| cme-miR394a | MELO3C022422T2 | MELO3C022422 | - | 1 | 1189-1208 | 1199 | 0 | 0.00971 |
| cme-miR394a | MELO3C022422T1 | MELO3C022422 | - | 1 | 1189-1208 | 1199 | 0 | 0.00971 |
| cme-miR395c | MELO3C006493T1 | MELO3C006493 | ATP sulfurylase 1 | 2.5 | 538-558 | 549 | 2 | 0.452 |
| cme-miR395c | MELO3C014280T1 | MELO3C014280 | ATP sulfurylase 1 | 2.5 | 383-403 | 394 | 2 | 0.452 |
| cme-miR395f | MELO3C006493T1 | MELO3C006493 | ATP sulfurylase 1 | 2.5 | 538-558 | 549 | 2 | 0.778 |
| cme-miR395f | MELO3C014280T1 | MELO3C014280 | ATP sulfurylase 1 | 2.5 | 383-403 | 394 | 2 | 0.778 |
| cme-miR396b | MELO3C024739T1 | MELO3C024739 | probable tocopherol O-methyltransferase | 3 | 408-429 | 419 | 0 | 0.0167 |
| cme-miR396b | MELO3C006174T2 | MELO3C006174 | - | 4 | 722-743 | 733 | 0 | 0.0208 |
| cme-miR396b | MELO3C012467T1 | MELO3C012467 | - | 3.5 | 1487-1507 | 1498 | 0 | 0.0179 |
| cme-miR396b | MELO3C007656T1 | MELO3C007656 | - | 3.5 | 926-947 | 937 | 0 | 0.0179 |
| cme-miR396b | MELO3C010417T1 | MELO3C010417 | - | 4 | 1796-1816 | 1807 | 0 | 0.0208 |
| cme-miR396b | MELO3C004605T1 | MELO3C004605 | DNA-directed RNA polymerase I subunit RPA12-like | 4 | 1406-1426 | 1417 | 0 | 0.0208 |
| cme-miR396b | MELO3C010786T1 | MELO3C010786 | - | 3.5 | 897-918 | 908 | 0 | 0.0179 |
| cme-miR396b | MELO3C006174T1 | MELO3C006174 | - | 4 | 269-290 | 280 | 0 | 0.0208 |
| cme-miR396d | MELO3C011072T1 | MELO3C011072 | E3 ubiquitin-protein ligase UPL1-like | 4 | 7356-7375 | 7366 | 2 | 0.966 |
| cme-miR396d | MELO3C024739T1 | MELO3C024739 | probable tocopherol O-methyltransferase | 4 | 408-429 | 419 | 0 | 0.0412 |
| cme-miR396d | MELO3C006174T2 | MELO3C006174 | - | 3.5 | 722-743 | 733 | 0 | 0.0119 |
| cme-miR396d | MELO3C012467T1 | MELO3C012467 | - | 4 | 1487-1507 | 1498 | 0 | 0.0412 |
| cme-miR396d | MELO3C007656T1 | MELO3C007656 | - | 4 | 926-947 | 937 | 0 | 0.0412 |
| cme-miR396d | MELO3C010786T1 | MELO3C010786 | - | 4 | 897-918 | 908 | 0 | 0.0412 |
| cme-miR396d | MELO3C006174T1 | MELO3C006174 | - | 3.5 | 269-290 | 280 | 0 | 0.0119 |
| cme-miR396d | MELO3C026387T1 | MELO3C026387 | - | 4 | 376-396 | 387 | 2 | 0.966 |
| cme-miR396e | MELO3C024739T1 | MELO3C024739 | probable tocopherol O-methyltransferase | 3 | 408-429 | 419 | 0 | 0.0134 |
| cme-miR396e | MELO3C014113T1 | MELO3C014113 | - | 2 | 2011-2030 | 2021 | 0 | 0.00412 |
| cme-miR396e | MELO3C008019T1 | MELO3C008019 | Heterogeneous nuclear ribonucleoprotein A1 | 3.5 | 210-230 | 221 | 2 | 0.618 |
| cme-miR396e | MELO3C006174T2 | MELO3C006174 | - | 4 | 722-743 | 733 | 0 | 0.013 |
| cme-miR396e | MELO3C007656T1 | MELO3C007656 | - | 3.5 | 926-947 | 937 | 0 | 0.0119 |
| cme-miR396e | MELO3C005757T1 | MELO3C005757 | luminal-binding protein 5 | 4 | 1146-1165 | 1156 | 2 | 0.651 |
| cme-miR396e | MELO3C010786T1 | MELO3C010786 | - | 3.5 | 897-918 | 908 | 0 | 0.0119 |
| cme-miR396e | MELO3C006174T1 | MELO3C006174 | - | 4 | 269-290 | 280 | 0 | 0.013 |
| cme-miR398a | MELO3C004454T1 | MELO3C004454 | - | 3 | 10-30 | 21 | 2 | 0.237 |
| cme-miR398b | MELO3C004454T1 | MELO3C004454 | - | 3 | 10-30 | 21 | 2 | 0.418 |
| cme-miR398b | MELO3C022138T1 | MELO3C022138 | - | 4 | 1605-1624 | 1616 | 2 | 0.19 |
| cme-miR408 | MELO3C008424T1 | MELO3C008424 | - | 2.5 | 104-124 | 115 | 0 | 0.00748 |
| cme-miR530a | MELO3C006469T2 | MELO3C006469 | - | 1.5 | 98-117 | 108 | 0 | 0.00897 |
| cme-miR530a | MELO3C006469T1 | MELO3C006469 | 40S ribosomal protein S15-4-like | 1.5 | 333-352 | 343 | 2 | 0.514 |
| cme-miR530b | MELO3C006469T2 | MELO3C006469 | - | 1 | 98-117 | 108 | 0 | 0.00971 |
| cme-miR530b | MELO3C006469T1 | MELO3C006469 | 40S ribosomal protein S15-4-like | 1 | 333-352 | 343 | 2 | 0.542 |

**Supplementary Table 4** Table of primer set of melon tissues qRT-PCR analysis, cme-miR393-OE transgenic plant line construction, and cme-miR393 target genes expression.

| miRNA | primers |
| --- | --- |
| cme-m1429-3p | TGCCAAAAGAGACTTGCCCTG |
| cme-miR159a | TTTGGATTGAAGGGAGCTCTA |
| cme-miR160a/b/c | TGCCTGGCTCCCTGTATGCCA |
| cme-miR162 | TCGATAAACCTCTGCATCCAG |
| cme-miR164c/d | TGGAGAAGCAGGGCACGTGCA |
| cme-miR166e | TCGGACCAGGCTTCATTCCTC |
| cme-miR167d/f | TGAAGCTGCCAGCATGATCTG |
| cme-miR168 | TCGCTTGGTGCAGGTCGGGA |
| cme-miR169c/d/o | TAGCCAAAGATGACTTGCCTG |
| cme-miR171f | TGATTGAGCCGTGCCAATATC |
| cme-miR2111a/b | TAATCTGCATCCTGAGGTTTA |
| cme-miR390a/b/c/d | AAGCTCAGGAGGGATAGCGCC |
| cme-miR393a/b/c | TCCAAAGGGATCGCATTGATC |
| cme-miR394a/b | TTGGCATTCTGTCCACCTCC |
| cme-miR395a/d/e/f | CTGAAGTGTTTGGGGGAACTC |
| cme-miR396b | TTCCACAGCTTTCTTGAACTG |
| cme-miR398a | TGTGTTCTCAGGTCGCCCCTG |
| cme-miR408 | ATGCACTGCCTCTTCCCTGGC |
| cme-miR477b | CTCTCCCTCAAAGGCTTCTG |
| cme-miR530b | TGCATTTGCACCTGCACCTT |
| cme-miR7129 | AGTCAAATCTAAACGATCGTGTAT |
| cme-miR854 | GATGAGGATAGTGAGGAGGAG |
| cme-miR858 | TCTCGTTGTCTGTTCGACCTT |
| U6-3 F | CCCTTCGGGGACATCCGATA |
| U6-3 R | CCATTTCTCGATTTGTGCGTGT |
| GAPDH1-P1 | GTCTTTCCGTGTTCCTACCG |
| GAPDH1-P2 | GACCTGTTGTCACCCACGAA |
| pre-miR393a-F | GACTAGT CCGGAGAAGTGGAGGGTAGA |
| pre-miR393a-R | AGTCTCCTCCTTCAAAAGAGTTTCA GGTACCc |
| 35S-R | TTCTTCTTGTCATTGAGTCGTA |
| 35S-F | CAGAAAGAATGCTAACCC |
| GMR-F | TTTCGGTCGTGAGTTCGGAG |
| GMR-R | CACTTCTTCCCGTATGCCCA |
| CmAFB2-F | TTTGAAGCGGATGGTCGTCA |
| CmAFB2-R | ACCTGCAATTGGCAGCAATG |
| TIR1-F | GTGTATCAGCACTGGAGCGT |
| TIR1-R | CAGCACCAAACTCAACCAGC |
| pre-miR393a | GAGGAAGCATCCAAAGGGATCGCATTGATCCCAACTTATATATTTACAGTTTTCTTTTCATTTTCTTTCTTCTACATAATTTTGGATCATGCTATCCCTTCGGATTCCTCCTTTA |

**Supplementary file 1** Sequences of Melon TIR1/AFB gene family.

>MELO3C025152 sp|O04197|COI1_ARATH Coronatine-insensitive protein 1 OS=Arabidopsis thaliana GN=COI1 PE=1 SV=1//0

ATGGAAGAACGGGATAGTAGTAGAGTAAATATGGGGATGCCGGATGTGATTCTTGGCTGTGTAATGCCCTATATTCAGGACCCCAAAGATCGTAACGCTATTTCTCAAGTTTGCCATAGATGGTACGAGCTTGATGCGCTTACTCGTAACCATGTTACCATAGCTCTTTGCTACACCACCACACCCGAACGGCTACGGCAACGGTTTGTTCATCTCGAGTCCTTGAAATTGAAGGGTAAACCAAGAGCTGCAATGTTTAATTTAATACCTGAGGATTGGGGTGGATATGTTACTCCATGGGTAAAGGAGATTGCTATTTCTTTCAACCGCTTGAAGTCTCTTCACTTCCGGCGTATGATTGTCGTAGATTCGGATCTGGAGCTTCTTGCTAGTGCACGAGGCCGTGTTCTTGTGTCGCTTAAACTCGATAAATGCTCTGGATTCTCCACCGATGGACTATTCCATATTGGACGCTCTTGCAGGAATTTGAAAACATTGTTTTTGGAAGAGAGCTCAATTCTGGAGAAAGATGGACAATGGTTACGTGAACTTGCTAGGAACAACACAGCGCTTGAGACGTTAAACTTTTACATGACAGATCTTACCCAAGTTAGATTTGAAGATCTTGAACTTATAGCCAGGAACTGTCGTTCCTTAATTTCTGTGAAAATTAGTGACTGTGAAATCCTTGATCTCGTGGGCTTCTTTCGTGCTGCTGGTGCTTTGGAGGAATTTTGTGGAGGTTCCTTCGGCTTCAATGACCAACCAGAGAGGTATGCAGGTATAGCATTGCCTCAGAACCTGCGTAATTTGGGTCTTACATACATGGGAAGAAGTGAAATGCCAATAGTTTTCCCTTTTGCAAGTCTTCTCAAGAAGTTGGATCTTTTGTATGCTCTGCTTCATACTGAAGATCATTGTACTTTGATTCAGAGATGCCCCAACTTAGAAATTCTTGAGACTAGAAATGTGATTGGAGATAGAGGATTAGAAGTTCTTGCTCGTCATTGCAAGAAACTGAAAAGGCTTAGAATTGAGCGAGGTGCGGACGAGCAGGGATTAGAAGATGAAGAAGGTCTTGTTTCACAAAGAGGATTGATTGCTTTGGCTCAGGGATGTCTGGAATTGGAATACTTAGCTGTATATGTGTCAGATATTACTAATGCATCTCTCGAGTGCATCGGCACATACTCAAAAAATCTCAGCGATTTTCGCCTTGTCCTGCTGGATCGAGAAGTACGGATAACGGATTTGCCACTTGACAATGGTGTTCAAGCTCTATTGAGAGGGTGTTCAGAGAAGCTGAAGAGATTTGCTTTATATCTTCGGCCTGGTGGATTGACAGACGTTGGTCTTGGTTATATAGGAAGGTATAGCCCAAATGTCAGATGGATGCTTCTTGGTTATGTGGGGGAATCTGATGCTGGGCTAGTGGAATTCTCTCGCGGCTGCCCTAGCCTACAGAAGCTTGAAGTTAGAGGATGTTGCTTTAGTGAACAAGCATTAGCAGAGTCCGTATTGAACCTAACTTCCCTCAGGTACCTATGGGTTCAAGGCTACAGAGGATCCTCTTCAGGCCGCGATCTTCTTGCAATGGCTCGCCGGTACTGGAACATCGAATTGATTCCGTCGAGACGAGTTGTTGTGCCTGATCAAGTTGGGGAGATGGTGGTGGCTGAACACCCTGCTCATATACTTGCATACTACTCCCTAGCAGGACCAAGAACTGATTTTCCAGAGAGCGTTGTACCGTTGGATTCGTAG

>MELO3C014799 sp|Q9LW29|AFB2_ARATH Protein AUXIN SIGNALING F-BOX 2 OS=Arabidopsis thaliana GN=AFB2 PE=1 SV=1//0

ATGAATTATTTTCCCGACGAGGTTTTAGAACATGTTTTCGATTATGTAACGTCTCACCGGGACCGGAACGCGGTGTCCTTGGTGTGCAAATTGTGGTACAGAGTTGATAGATTTAGTAGACAGAAAGTGTTCGTCGGAAATTGCTATTCGATCACGCCGGAGAGGGTAATCGGAAGATTCCCCTGTGTTAAATCCCTAACCCTAAAGGGAAAGCCCCATTTCGCTGATTTCAATTTGGTCCCTCATGATTGGGGTGGCTACGTTTACCCTTGGATCCAAGCCTTTGCGAAGCGCAGGATTTCCCTCGAGGAGCTTCGTTTGAAGCGGATGGTCGTCACCGACGACAGTCTCGAGCTTCTTTCTCGATCCTTCCCCAATTTCAAGTCCCTTCTGCTTTTCAGCTGTGAGGGATTTACCACCAATGGCCTTGCTGCCATTGCTGCCAATTGCAGGTTTCTGAGGGAGCTTGACCTGCAAGAGAATGAAATTGATGACCATAGTAATTACTGGGTTAGCTGCTTCCCGGAGAGCTGCACGTCGCTTGTCTCCCTGAATTTTGCTTGCCTCAGAGGAGAAGTGAATCTGGGTGCTCTTGAGAGGCTTGTAGCAAGATCTCCTAACCTCAAGAGTTTGAGGCTGAACCGTGCAGTGCCTATTGAAACCTTGCAAAATATATTGGCAAATGCTCCTCAACTTGTGGACTTGGGCACCGGGTCTTACGTTCATGATCGAGATTCTGAGATCTATGACAATCTCAAGAACACCCTTCTGAAATGCAAATCAATCAGGAGTTTATCTGGTTTTTTAGATGTTTCTCCTTGCTGCCTGGCCTCCATTTACCCAATTTGCTCCAATTTGACATCCTTGAACCTGAGCTATGCTCCTGGGCTTCATGGCAATGAGCTCATAAAGGTCATTCAGTATTGCGAGAGACTTCAACGATTGTGGATTCTGGATGGTATTGGAGACAAAGGACTGGAAGTTGTTGCTTCATCTTGTAACGAATTGCAGGAATTGAGGGTTTTCCCATCTGACCTCTCCGGGGCTGGTAATGTTGCTGTCACAGAAGAAGGTTTGGTAGCCATATCGAAGGGGTGCCCCAAACTTCATTCGATATTGTACTTCTGCCATCAGATGACAAATGCTGCCCTTGTAACTGTAGCAAAGAACAACCCAAACTTCATACGTTTTAGGCTGTGCATCCTCGACCCCACAAAACCAGATCCTGTTACTGGGTATCCACTGGATGAAGGTTTTGGGGCGATTGTTCAAGCGTGTAAAGGTCTGAGACGTTTATCTCTCTCAGGCCTTCTTACGGATCAGGTATTTTATTACATTGGTGAATATGCAAAGCATCTAGAAATGCTTTCGTTAGCATTTGCTGGGAACAGTGACAAGGGAATGATTCACGTATTGAGTGGTTGCAAGAAACTTCGCAAGCTCGAGATCATGGACAGCCCGTTTGGTGACATGGCACTTCTGCAGGACGTTGGGAAGTATGAAACAATGCGATCCCTTTGGATGTCGTCCTGTGAGATTACTCTTGGTGGCTGCAAGACATTAGCAAAGAAGATGCCGAGGCTGAACGTGGAGATCATCAACGAGAATGATCAGTTGGGATTCTGCCGCAATGTGGATATGATCAACGAGAATGGTCAGTCAGAAGTCTGCCGTGATGATGAGCAGAAAGTAGGGAAAATGTATCTGTACCGTACATTAGTAGGACCAAGGAAAGATGCTCCTAAGTTTGTATGGACATTGTAG

>MELO3C003538 sp|Q9LTX2|TIR1L_ARATH Transport inhibitor response 1-like protein OS=Arabidopsis thaliana GN=At5g49980 PE=2 SV=1//0

ATGAGAGTGGACAAAGCAGAAATGTCTGAAGACGAGGATCGGGCTCAATCTCTAGATCTGGGTATCGAGGCCGCCGCCGAATCCGCTAACAAAACCCGTAATTGTAGCGCCGGCGACGGTGAGGAGGTTGGTGGGTCTGGCTCAGTGGAGAACATTCTCCATAATGTTCTTGAGAACGTGCTTCATTTCCTCACTTCACGACGTGACCGGAACGCGGCTTCACTTGTTTGCAAGTCCTGGTATCGTGTTGAAGCCCTCACTCGATCCGAACTCTTCATTGGAAATTGCTATGCCGTCTCACCTCGCCGAGTTACGTCGCGGTTTAATCGAGTTCGGTCAGTGTCGATCAAAGGGAAGCCTCGGTTTGCTGATTTCAACCTGATGCCGCTCAATTGGGGGGCTCACTTTACTCCATGGGTGGCGGCCATGGCTAAGTCGTACCCTTGGCTCGAGAGAGTCTATTTGAAGCGCATGTCTGTGACTGACGACGATCTGGCTCTGCTTGCTGACTCTTTTCCCGGCTTTAAGGAGCTTGTGTTGTTTTGCTGTGAAGGATTTGGCACCAGTGGTATTGCTGTTGTTGCCACCAGATGCAGGCACCTTCGAGTACTTGATCTGATTGATTCTGATGTAAAAGATGATGAAGTGGATTGGATATCTTGTTTTCCTGAGAAGGAAACTTGTCTTGAATCTCTGATCTTTGAATGTGTAGAGTGGCCTATTAATTTTGAGGCACTGGAGAGGTTAGTGAGTAGATCCCCATCCTTGAAGAAACTTGGGGTGAATCGTCACGTTTCCATTGCTCAGTTATACCAATTGATGATTCAGGCTCCTCGTTTGACACATCTGGGAACCGGTTCATTCAATACATCAGAGGCTGTGATCCATGGCGAGTCCGAGCCTGATTATGCCTCGGTTTTTGCTGCTTGCAATTCCTTAGTTTGTCTCTCTGGATTCAAGGATGTTTTGCCCGATTACCTACCTTGTATCTATCCAGTTTGTGCTAACCTCACCACTCTGAACTTGAGCTATGCAAATATAACTCCTGAACAGCTTAAACCAGCTATAAGACACTGCCACAAGCTCCAGACTTTCTGGGCTCTTGATTCAATATGCGACGAAGGACTTCAGGCTGTTGCTTCAACTTGCAAGGAACTGCGAGAACTTAGGGTTTTCCCTGTTGATCCCCAAGAAAATGGCGAAGGCCCCATTTCTGAAGTGGGCTTCCAGGCAATATCTGAAGGTTGCAGGAAATTGCAATATATTCTCTACTTTTGCCAGAGAATGACTAATGCAGCCGTAGTAGCTATGTCAGAGAACTGTCAAGATCTTGTGGTGTTTAGACTGTGTATTATGGGACGACACCAGCCAGACCATAAAACTGGAGATCCAATGGACGAAGGATTTGGTGCCATTGTTATAAACTGTAAGAAGCTAACTAGGCTTGCAATATCTGGATTGTTGACGGATCGTGCTTTCAGCTATATCGGGAAGTATGGGAAGCTGGTACGCACCCTGTCAGTTGCTTTTGCTGGAAACAGTGATTTGGGATTGAAGTATGTGCTTGAGGGCTGTCATAGATTGCAAAAACTTGAGATTAGAGATAGCCCTTTTGGGGATATAGCTTTACATTCTGGTTTACATCATTACTACAATATGAGATTCCTCTGGATGTCAGACTGTAAATTATCTCGCCAGGGCTGCCAAGTGGTTGCCAAAGCAATGCCTCACTTGGTGGTTGAAGCGATGAGAAATGATATCGAGGAAGTGGATTCTCATCCTCAGGTGGAGGATTTGGACAACCATGTTCGCTTGTTATACATGTATCGATCTCTTGAAGGGCCAAGGGATGATGCTCCGGAATTTGTAGACATCTTATAG

>MELO3C003539 sp|Q9LTX2|TIR1L_ARATH Transport inhibitor response 1-like protein OS=Arabidopsis thaliana GN=At5g49980 PE=2 SV=1//0

ATGAGAGTGGATCAAGCTGAAGAAATGTCCGAAGACGAAGATCGATCTTTAACTTCGGATCTAGTTGGTGGTGGTGGTGGAGGTGGTGATGCCATAGCTGAATCTGCTAGTAAAACTCGCAATTGCACAGGGTGTTCTAGTGATGTTACTGCTTCTGGTTCAAATACACTTCATAATGTTCTTGAGAACGTGCTTCACTTTCTAACTTCACGACGTGACCGGAACGCGGCTTCACTTGTTTGCAAGTCATGGTATCGAGTTGAAGCTCTTACTCGATCCGACCTTTTCATTGGGAATTGTTATGCGGTATCCCCTCGTCGAGTTACGTCGCGGTTTAATCTAGTTCGGTCAGTGAGTATTAAAGGGAAGCCTCGGTTTGCTGATTTCAACCTGATGCCGATCAATTGGGGGGCTCACTTTACTCCATGGGTGGCGGCTATGGCTAAGTCATACCCTTGGCTTGAGAGAGTATATTTGAAGCGTATGTCTGTGACTGATGACGATCTGGCTCTGCTTGCTGACTCTTTTCCTGGCTTTAAGGAGCTTGTGTTGTTTTGCTGTGAAGGATTTGGCACCAGTGGTATTGCTGTTGTTGCCGCCAGATGCAGGCACCTTCGAGTACTTGATCTGATTGAATCTGACGTAGCGGATGATGAAGTGGATTGGATATCTTGTTTCCCTGAGAAGGAAACTTGTCTTGAATCTCTGATCTTTGATTGTGTAGAGTGGCCTATTAATTTTGAGGCACTGCAGAGGTTGGTGAGTAGATCCCCATCGTTGAAGAAGCTTGGGGTGAATCGTCATGTTTCCATTACTCAGCTGTACCATTTGATGATTCGGGCTCCACATTTAACGCATCTGGGAACTGGTTCATTTAGTACTTCAGAGGCTGTGGTTCATGGGGACTCAGAGCCTGATTTTACCTCTGCTTTTGCTGCTTGCAAATCCTTAGTTTGTCTTTCTGGATTCAAGGACATTTTGCCTGATTATCTACCTTGTATCTATCCAGTTTGTGCTAATCTCACCACTCTGAACTTGAGCTTTGCAAATATAACCCCTGAACAACTCAAACCAGTCATAAGTCACTGCCACAAGCTCCAAACTTTCTGGGCTCTTGATTCGATATGTGACGAAGGACTTCAGGCTGTTGCTTCAACTTGCAAGGAACTGCGAGAACTTAGGGTTTTCCCTGTTGATCCCCGAGAAGATGTCGAAGGCCCCATTTCTGAAGTGGGCTTCCAGGCAATATCTGAAGGTTGCAGGAAATTGCAATATATTCTATACTTTTGCCAGAAAATGACCAATGCAGCCGTAGTAGCTATGTCACAGAACTGCCAAGATCTTGTGGTGTTTAGACTGTGTATTATGGGACGACACCAGCCAGACCATAAAACCGGAGATCCAATGGATGAAGGATTTGGTGCCATTGTTATTAACTGTAAGAAGCTAACTAGGCTTGCAATATCTGGATTGTTGACGGATCGTGCTTTCAGCTATATTGGGAAGTATGGGAAACTGGTACGCACCCTGTCAGTTGCTTTTGCTGGAAACAGTGATTTGGGGTTGAAGTACGTGCTTGAGGGCTGTCATAGATTGCAAAAACTTGAGATAAGAGATAGCCCATTTGGTGATGGAGCCTTGCGGTCTGGTTTACATCATTACTACAATATGAGATTTCTCTGGATGTCAGCTTGTAAATTGACTCGCCAAGGCTGCCAAGAGGTTGCTAGAGCGATGCCTCACTTAGTGGTTGAGGTGATGAAGAGTGACGAGGAAATTGAAAACGATAGTCAGGTGGAGGGTATGGAGGACCATGTTCAGGTGCTATATATGTATAGATCTCTTGAGGGACCAAGGAATGATACTCCAAAGTCTGTTGACATCCTATAG

>MELO3C007323 sp|Q9LPW7|AFB3_ARATH Protein AUXIN SIGNALING F-BOX 3 OS=Arabidopsis thaliana GN=AFB3 PE=1 SV=1//0

ATGAGTTCGAAGAGGAGGAAAGGGTCAGGTGACTCGGATGAGTCGAATCGGGCTGGTTCAATTTTCCCAGATGAGGTATTGGAAAGAGTTTTGAGTTTGGTGAAATCCCACAAAGATAGAAGCTCTGTGTCTTTGGTTTGTAAAGATTGGTTCAACGCTGAGCGGTGGTCTCGAACCCATGTTTTCATTGGGAATTGTTACTCTGTTTCGCCGGAAATTGTAATCCGACGGTTCCCGAACATTCGTAGTGTGACATTGAAAGGGAAACCTAGGTTTTCGGATTTCAATTTGGTGCCTCCAAATTGGGGAGCTGATGTTCATTCATGGCTTGTTGCTTTCGCTTCAAAATACCCAATTCTTGAAGAATTGAGGCTCAAAAGAATGACTGTAACGGACGAAAGCTTGGAGTTTTTGAGTCGTTCCTTTCCAAATTTCAAAGCCCTATCAATGATGAGTTGCGATGGCTTCAGCACCGATGGCCTTGCCGCCATTGCCACTAACTGCAAGAACTTGACTGAGCTTGACATACTTGAGAATGACATTAATGACAAGAGTGGAAATTGGCTGAGTTGCTTCCCAGATACTTTGAAATCTCTGGAAGTACTCAACTTTGCAAGTTTGAATAGTGATGTTAGTTTTGAGGCCCTTGAGAAGCTGGTAAGAAGGTGCAAATCGTTGAAGGTTTTGAAGGTCAATAGGAATATAAACTTGGAACAATTGCAGAGGCTGCTTGTTCACACTCCTCAGTTAACGGAGTTAGGCATGGGCTCTTTTTCGCAAGAGATCACTCTTCGGCAGTATTGCGACCTTGAAGAGGCTTTTAAAAGTTGCAAGAATCTGCACACTCTCTCTGGTTTGTTGGAGTCGACAGTGTTGTATCTCCAAGTTCTTTTTCCTGCCTGTGCAAATATAACTTTCTTAAATCTGAGCTATGCTATTCTACACGGTGGTGAACTTGCTGGACTCCTTTCCCACTGCCCTGTTCTTCGCCGTCTTTGGGTTTTAGACACAGTTGAAGACAAGGGCCTGAAGGCCGTTGGACTGAGTTGCCCCTTGCTCGAGGAACTCCGTGTATTTCCAGCACATCCGTTTGCTGACAATTTAGTCCATGGTGTAACTGAGTCGGGGTTTCTCGCAGTCTCTTATGGCTGTCGTAAACTCCGTTACGTTCTCTACTTTTGCCATCAGATGACGAATGAGGCTGTGGCCACCATTGTGCAGAACTGCCCAGATTTCACCCATTTTCGTCTCTGCATTATGAATCCCCACCAACCAGACTATCTTACAAAGCAACCAATGGATGAGGCCTTTGGTGCCGTAGTGAAAACTTGTTCTAAGCTGCGTAGACTCGCCATTTCAGGCTTGTTAACGGACTTAACATTTGAATACATCGGGAAATATGCAAAAAACTTGGAAACTCTTTCAGTGGCTTTTGCTGGAAGCACTGACTGGGGAATGCAGTGTGTTATGAGTGGTTGTCCAAAGCTGAAGAAGCTGGAGATCAGAGACAGCCCATTTGGAAATGCAGCTTTGCTTTCTGGTTTGGAGAGGTATGAATCCATGAGATCACTATGGATGTCGGCCTGCAAGGTGACGATGAATGGGTGTCGAGTCTTGGCGAAGCAGGTGCCTAGGTTGAATGTTGAGGTCATTAAGGATGATGGAAATGACGAATGTGAGGCTGAAAGTGTTTATGTATACCGCTCTGTCGCAGGTCCACGTAGAGACGCCCCACCTTTTGTTGTCACCCTCTAA

>MELO3C015898 sp|Q570C0|TIR1_ARATH Protein TRANSPORT INHIBITOR RESPONSE 1 OS=Arabidopsis thaliana GN=TIR1 PE=1 SV=2//0

ATGCTGAGAATGGCGAGTACGTTTCCAGAAGATGTGTTGGAGCATGTGTTTTCGTTTATACAGTGTCATAAAGACAGGAACTCGATTTCTATGGTGTGTAAATCTTGGTATGAGATTGAGAGATGTTGTAGGAAGAGGGTTTTTGTTGGGAACTGCTATGCGGTGAGTCCTCAGACTGTAATCAGGCGGTTTCCGGATGTGAGATCTGTGGAGTTGAAAGGGAAGCCGCACTTTGCGGATTTTAGTTTGGTGCCTGATGGTTGGGGTGGGTATGTTTATCCATGGATCCTGGCTATGGCTTCTGCGTATCCTTTGTTGGAGGAGATCAGGTTGAAGAGAATGGTGGTTACGGATGAGAGTTTGGAGCTCATTTCCAAGTCTTTTAAGAATTTTAAGGTTCTTGTGTTGATGACTTGTGAAGGGTTCAGTACTGATGGGCTTGCGGCCATTGCTGCTAATTGCAGGAATTTGAAAGTATTGGACTTACGAGAGAGTGATGTGGAAGATCTGAATGGGCATTGGCTCAGCCATTTTCCTGATACATATACGTCATTGGTATCTCTCAATATTGCTTGTTTGGGGTCTGAGGTGAGTGTATCAGCACTGGAGCGTCTAGTGGACAGGTGTCCAAACTTAAGGACACTGCGTCTCAATCGTCCAGTTCCCCTTGATAGGCATGCCAACCTACTTCGTCGTGCCCCTCAGCTGGTTGAGTTTGGTGCTGGATGCTATATGGCTGACTTGAGATCTGAAGTTTTTTCGAGCTTAACTGGGGCATTTACAAGCTGCACAGAATTGAAGAGTCTGTCAGGATTTTGGGATGTTGTCCCTGCATACCTTCCTTCTGTTTATCCAACATGCTCTCGGCTAACATCCTTAAACTTAAGCTATGCTACAATTCAATGTGACGATCTTACAAAGCTTATCAGTCAATGTCATAATTTACAGAAGCTTTGGGTGCTGGACTTCATTGAGGATTCTGGCCTTGAAGCTGTTGCAGATACCTGCAAAGATCTTAGAGAATTAAGAGTGTTTCCATCCGAACCGTATGGGCAGGAACCAAATGTGTCTTTGACAGAACAGGGCCTCGTCTCTGTATCTGAAGGTTGCCCAAAGCTCCAATCTGTTCTGTACTTTTGTCGTCAAATGACAAATGCTGCCTTAGTGACCATTGCCAGGAATCGTCCAAACATGACCAGATTTCGTCTATGTATTCTGGAGCCACGGACTCCCGATTACCTTACGGATCAGCCACTTGATGTAGGTTTTGGGGCAATTGTTGAGTATTGCAAAGATCTCCAGCGCTTTTCCCTTTCTGGCCTTCTTACGGATCGATGTTTCGAATATATAGGGACTTATGGTAAAAAGCTAGAGATGTTATCTGTGGCATTTGCAGGCGATAGTGATTTAGGTCTTCATCATGTGTTGTCTGGATGTGATAGTCTTCGCAAGCTGGAGATCAGGGACTGTCCTTTTGGTGATAAAGCTCTTTTAGCCAATGCTGCAAAGCTCGAGACAATGCGATCCCTTTGGATGTCTTCTTGCTCAGTGAGTTTTGGAGCATGCAAATTGTTAGCTCAGAAGTTTCCCCAACTCAATGTCGAAGTTATAGATGAGAGGGGTCCTCCAAATACCAGGCCCGAAAGTTGTCCTGTGGAGAGGTTATATATATACCGAAGTGTCGCTGGGCGTCGGTTAGATATGCCTGGTTTTGTGTGGACAATGGACGGAGATGCTCCTCTAAAGCTTACTTGA
